# Toward tuberculosis elimination by understanding epidemiologic characteristics and risk factors in Hainan Province, China

**DOI:** 10.1186/s40249-024-01188-2

**Published:** 2024-02-27

**Authors:** Changqiang Zhou, Tao Li, Jian Du, Dapeng Yin, Xiujun Li, Shixue Li

**Affiliations:** 1 Department of Biostatistics, School of Public Health, Cheeloo College of Medicine, Shandong University, 44# Wenhuaxi Road, Lixia District, Jinan, Shandong 250012 People’s Republic of China; 2 National Center for Tuberculosis Control and Prevention, Chinese Center for Disease Control and Prevention, Beijing, People’s Republic of China; 3 Clinical Center On TB Control, Beijing Chest Hospital, Capital Medical University/Beijing Tuberculosis and Thoracic Tumor Research Institute, Beijing, People’s Republic of China; 4 Hainan Center for Disease Control and Prevention, Haikou, Hainan 570203 People’s Republic of China; 5 Research Center for Tuberculosis Control, Shandong University, Jinan, Shandong People’s Republic of China

**Keywords:** Tuberculosis, Elimination, Hainan Province China, Epidemiological characteristics, Geographically, Temporally weighted regression

## Abstract

**Background:**

The disease burden of tuberculosis (TB) was heavy in Hainan Province, China, and the information on transmission patterns was limited with few studies. This atudy aims to further explore the epidemiological characteristics and influencing factors of TB in Hainan Province, and thereby contribute valuable scientific evidences for TB elimination in Hainan Province.

**Methods:**

The TB notification data in Hainan Province from 2013 to 2022 were collected from the Chinese National Disease Control Information System Tuberculosis Surveillance System, along with socio-economic data. The spatial–temporal and population distributions were analyzed, and spatial autocorrelation analysis was conducted to explore TB notification rate clustering. In addition, the epidemiological characteristics of the cases among in-country migrants were described, and the delay pattern in seeking medical care was investigated. Finally, a geographically and temporally weighted regression (GTWR) model was adopted to analyze the relationship between TB notification rate and socio-economic indicators. The tailored control suggestions in different regions for TB elimination was provided by understanding epidemiological characteristics and risk factors obtained by GTWR.

**Results:**

From 2013 to 2022, 64,042 cases of TB were notified in Hainan Province. The estimated annual percentage change of TB notification rate in Hainan Province from 2013 to 2020 was − 6.88% [95% confidence interval (*CI*): − 5.30%, − 3.69%], with higher rates in central and southern regions. The majority of patients were males (76.33%) and farmers (67.80%). Cases among in-country migrants primarily originated from Sichuan (369 cases), Heilongjiang (267 cases), Hunan (236 cases), Guangdong (174 cases), and Guangxi (139 cases), accounting for 53%. The majority (98.83%) of TB cases were notified through passive case finding approaches, with delay in seeking care. The GTWR analysis showed that gross domestic product per capita, the number of medical institutions and health personnel per 10,000 people were main factors affecting the high TB notification rates in some regions in Hainan Province. Different regional tailored measures such as more TB specialized hospitals were proposed based on the characteristics of each region.

**Conclusions:**

The notification rate of TB in Hainan Province has been declining overall but still remained high in central and southern regions. Particular attention should be paid to the prevalence of TB among males, farmers, and out-of-province migrant populations. The notification rate was also influenced by economic development and medical conditions, indicating the need of more TB specialized hospitals, active surveillance and other tailored prevention and control measures to promote the progress of TB elimination in Hainan Province.

**Supplementary Information:**

The online version contains supplementary material available at 10.1186/s40249-024-01188-2.

## Background

Tuberculosis (TB), a chronic infectious disease caused by a bacterium called *Mycobacterium tuberculosis*, is one of the significant public health issues worldwide. According to the report of World Health Organization (WHO), the TB bacterial infection affected around one-third of the global population. Annually, over 10 million people contracted TB, resulting in approximately 1.5 million deaths, making it one of the top ten leading causes of mortality [1]. Particularly in low-income and middle-income countries, the spread and prevalence of TB were more severe under conditions of dense population, high internal mobility, and low-level healthcare facilities and services [1, 2]. China was one of the highest TB burden countries, particularly in western and rural regions, the incidence and mortality rates of TB were notably elevated [3, 4]. Despite some achievements gained from the TB control programme, TB elimination is still one of the important public health issues in the world and China [5, 6].

Hainan Province is a tropical island in China with an average annual temperature of 23–29 ℃ and high humidity. Such environment created a conducive condition for the transmission of TB. Additionally, as one of the most famous tourist attractions in China, its frequent mobility of cross-border personnel increases the difficulties in the provincial TB prevention and control programme of Hainan Province. The TB epidemic in Hainan Province has always been serious, especially in economically undeveloped rural areas and specific populations such as migrant workers, characterized by a high incidence of TB [7–10]. A study in 2021 showed that the annual average notification rate of TB in Hainan Province was 85.15/100,000 [10]. Meanwhile, the TB mortality in Hainan Province was also high, accompanied by a substantial disease burden [11]. Therefore, TB elimination in Hainan Province has been difficult. The WHO has developed the Global Strategy to End TB, which set out a long-term vision for the global initial elimination of TB by 2035 (Incidence Rate < 10/100,000) and the complete elimination of TB by 2050 (Incidence Rate < 1/100,000) [6, 12]. To achieve the goal of TB elimination set by WHO, further prevention and control measures are needed in Hainan Province.

Understanding the spatial–temporal distribution of TB patients in a typical region can reveal the epidemiological characteristics in different geographical locations and help to specify corresponding TB elimination measures [13]. Previous reports provided some relevant information, but further exploration on spatial–temporal distribution of TB patients distribution is needed to provide more evidences to promote the comprehensive and effective TB prevention and control measures [14–16]. Furthermore, although the incidence of TB in Hainan Province has shown a downward trend in recent years, it is still at a relative high level. Exploring the impact factors on the prevalence of TB in Hainan Province can provide a scientific basis for designing more effectively TB control programme [17].

Up to now, there are some studies on the analysis of risk factors of TB, but most of them only relied on traditional statistical methods without considering spatio-temporal factors [5, 18]. Traditional statistical methods are usually based on regression and correlation analysis method, these methods mainly through comparing the differences between different groups to analyze the relationship between TB and risk factors, but often ignored the space and time dimensions. Space–time dimension changes are often not through the traditional statistical methods to capture, ignore the spatial and temporal correlation are likely to lead to bias the results [19, 20]. Given variations in economic development, medical infrastructure, and cultural practices across diverse regions in Hainan Province, it is imperative to perform spatio-temporal analysis techniques to explore the influencing factors of TB distribution and transmission. Geographically and temporally weighted regression (GTWR) analysis is a statistical analysis method that combines spatio-temporal factors and geographical location, which can reveal the factors affecting the distribution of TB more accurately. Compared with the traditional statistical methods, GTWR method can be used to model the spatial–temporal weight of TB data and analyze the spatial–temporal heterogeneity of TB cases. It can help identify hotspots and vulnerable areas, allowing for proactive intervention and allocation of resources to achieve TB elimination.

TB remains as a serious public health problem in Hainan Province of China. To better cope with TB elimination, it is necessary to further analyze TB distribution and transmission in Hainan Province, so as to enhance the understanding of the local TB. Therefore, this investigation is performed to explore the epidemiological characteristics and identify possible risk factors of TB transmission in Hainan Province by using multi-approaches including descriptive analysis, spatio-temporal distribution feature analysis, and GTWR. This endeavor aims to enhance the understanding of TB transmission patterns, which provide evidences for further improving the TB elimination programme in Hainan Province.

## Methods

### Data collection

The TB notification data in Hainan Province from 2013 to 2022 were collected from the Chinese National Disease Control Information System Tuberculosis Surveillance System, which recorded detailed information of TB patients, including outpatient information, case information, treatment, and supervision, as well as information on TB planning and management at county level. Population and socio-economic data were collected from the Hainan Statistical Yearbook (https://www.hainan.gov.cn/hainan/tjnj/list3.shtml).

Based on international codes of the current residence and household notification area of TB cases, all cases were divided into cases within county of the district, between districts and counties within the city, between cities in Hainan Province, and outside Hainan Province.

Based on previous research and literature, the interval between the appearance of TB symptoms in patients and their first visit to any medical institution was defined as the patient delay duration in seeking medical attention [21]. Due to data limitations, this study used the interval day(s) between the appearance of TB symptoms and the first visit to a TB-designated medical institution as a substitute and defined patient delay as duration of patient delay > 14 days [21]. Patients with a duration of patient delay less than 0 days were excluded when studying the patient delay.

### Statistical analysis

#### Descriptive analysis

The monthly and yearly notification rates of TB in Hainan Province from 2013 to 2022 were described, and the trend over time was analyzed. The estimated annual percentage change (EAPC) in notification rates for different genders and age groups was calculated. If the EAPC is greater than 0 and the confidence interval does not span 0, it indicates a continuing upward trend of the disease; if it is less than 0 and the confidence interval does not span 0, it indicates a continuing downward trend. If the confidence interval spans 0, it suggests no statistical significance in the trend of the disease [22]. The specific formula was as follows:

$${\text{ln}}\left(Rate\right)=\alpha +\beta *year+\varepsilon$$

$$EAPC\left(95\% CI\right)=100*\left({e}^{\left(\beta \pm 1.96*SE\right)}-1\right)$$

where Rate referred to the TB notification rate, $\alpha$ was the intercept, $\varepsilon$ was the residual, year was the calendar year, EAPC referred to the estimated annual percentage change, $\beta$ and $SE$ were the coefficient and standard error of the year derived from linear regression, and 95% *CI* referred to the 95% confidence interval. The Joinpoint model was used to find the “inflection point” or “turning point” of TB in Hainan Province to determine the point of trend change and the form of change. The model Settings were detailed in the Additional file 1.

With cities and counties as units, maps of the spatial distribution of TB notification rates and related influencing factors were drawn. Using information such as age, gender, and occupation, the population distribution characteristics of TB cases were analyzed. The migration of TB cases reported in Hainan Province and epidemiological characteristics such as diagnosis time, age, gender, and occupation of migrant cases from outside the province were described.

This study explored the TB prevention and control situation in Hainan Province by describing the discovery methods and registration composition of TB patients in the region. The patient delay duration in seeking medical attention (represented by the median, lower quartile, and upper quartile) and the number of patients with patient delay were described.

#### Spatial analysis

By using data at cities and counties levels, we undertook global and local spatial autocorrelation analysis of TB notification rates in Hainan Province.

Global spatial autocorrelation analysis was utilized to investigate whether there was a particular spatial aggregation or dispersion characteristic among the TB notification rates across different cities and counties in Hainan. In this study, we employed Moran’s *I* statistic to reflect spatial autocorrelation, with the specific calculation formula as follows:

$$I=\frac{n}{{\sum }_{i=1}^{n}{\sum }_{j=1}^{n}{\omega }_{i,j}}\frac{{\sum }_{i=1}^{n}{\sum }_{j=1}^{n}{\omega }_{i,j}{z}_{i}{z}_{j}}{{\sum }_{i=1}^{n}{z}_{i}^{2}}$$

where $n$ represented the number of observation units, ${\omega }_{i,j}$ signified the spatial weight between elements $i$ and $j$, ${z}_{i}$ and ${z}_{j}$ were the differences between the observed values of elements $i$ and $j$ and the average value X of the research area. The Z-statistic was calculated to test the statistical significance of spatial autocorrelation with the formula:

$$Z=\frac{I-E[I]}{\sqrt{V[I]}}$$

where $E\left[I\right]$=$-1/\left(n-1\right)$ and $V\left[I\right]$=$E\left[{I}^{2}\right]-E{\left[I\right]}^{2}.$ If Z > 1.96 or Z <  − 1.96, this indicates that Moran’s *I* index is statistically significant. Moran’s *I* ranges from − 1 to 1, with Moran’s *I* > 0 indicating positive spatial correlation—the larger the value, the more apparent the spatial correlation. Conversely, Moran’s *I* < 0 indicates negative spatial correlation—the smaller the value, the greater the spatial variance. If Moran's *I* = 0, it suggests that the observed values are spatially random [17, 23].

Local spatial autocorrelation was used to study the correlation between observed values and these in adjacent areas. The correlation results have four levels: high–high, low–high, high–low, and low–low. High-high signifies high notification areas have neighboring regions with high notification rates. Low–high means low notification areas have neighboring regions with contrary high notification rates. High–low denotes high notification areas that have neighboring regions with contrastingly low notification rates. Low–low suggests low notification areas have neighboring regions with similar low notification rates. The results of local spatial autocorrelation were expressed using LISA cluster maps [24].

#### Influence factors analysis

We investigated the relationship between the TB notification rates and socio-economic indicators in different cities and counties in Hainan Province. Based on prior research and local characteristics, selected independent variables included the proportion of rural population (%), GDP per capita (Yuan, the unite of Chinese currency), number of medical institutions per 10,000 people, and number of health personnel per 10,000 people. These indicators served as essential reflections of regional population structure, economic development level, and the availability of medical and health services in the region.

Geographically and temporally weighted regression (GTWR) model was used in the study following the methods of Huang et al. in which the regression parameters of the independent variables vary with changes in spatio-temporal location [19, 20]. GTWR is a statistical method that combines spatio-temporal weights to solve the problem of spatio-temporal heterogeneity in data. The basic principle is to consider the dependence of data in space and time, and to conduct regression analysis by adopting different weights in the space and time dimensions. Traditional regression models assume that the spatial and temporal distribution of data is uniform and ignore the heterogeneity of data in space and time. GTWR captures the spatio-temporal heterogeneity of data by using spatio-temporal weights.

The GTWR model established in this study was as follows:

$${Y}_{i}={\beta }_{0}\left({u}_{i},{v}_{i},{t}_{i}\right)+{\sum }_{k=1}^{m}{\beta }_{k}\left({u}_{i},{v}_{i},{t}_{i}\right){X}_{ik}+{\varepsilon }_{i}$$

where ${Y}_{i}$ denoted the TB notification rate of the *i*th city or county, $\left({u}_{i},{v}_{i}\right)$ represented the geographical coordinates of the *i*th city or county, ${t}_{i}$ signified the observation year, ${X}_{ik}$ indicated the kth explanatory variable of the *i*th city or county, ${\varepsilon }_{i}$ was the error term of the model, ${\beta }_{0}\left({u}_{i},{v}_{i},{t}_{i}\right)$ was the regression constant for the *i*th city or county, and ${\beta }_{k}\left({u}_{i},{v}_{i},{t}_{i}\right)$ stands for the regression coefficient of the *k*th explanatory variable for the *i*th city or county.

The regression coefficients in model fitting were calculated by the following formula:

$$\widehat{\beta }\left({u}_{i},{v}_{i},{t}_{i}\right)={\left[{X}^{T}W\left({u}_{i},{v}_{i},{t}_{i}\right)X\right]}^{-1}{X}^{T}W\left({u}_{i},{v}_{i},{t}_{i}\right)Y$$

where $W\left({u}_{i},{v}_{i},{t}_{i}\right)$ denoted the weight matrix of the *i*th city or county sample point, which reflected the influence weight of other cities or counties on this sample point. First, the spatial distance between the city or county sample points was calculated by the Euclidean distance formula ${d}_{ij}^{S}=\sqrt{{\left({u}_{i}-{u}_{j}\right)}^{2}+{\left({v}_{i}-{v}_{j}\right)}^{2}}$, and similarly, the temporal distance between the city or county sample points was calculated by ${d}_{ij}^{T}=\sqrt{{\left({t}_{i}-{t}_{j}\right)}^{2}}$. Since the measurement units of time distance and space distance were different, which can affect the results, the spatio-temporal distance calculation formula was ${\left({d}_{ij}^{ST}\right)}^{2}=\lambda {\left({d}_{ij}^{S}\right)}^{2}+\mu {\left({d}_{ij}^{T}\right)}^{2}$.

The weight function in this study was chosen to be an adaptive Gaussian function, with the specific weight calculation formula as follows:

$${W}_{ij}=exp\left(-\frac{{\left({d}_{ij}^{ST}\right)}^{2}}{{h}^{2}}\right)$$

where *h* was a parameter in the weight function that indicates the decay of spatio-temporal distance and represents the degree of local calibration smoothing. The optimal bandwidth h was determined by the corrected Akaike’s information criterion (AICc).

The fitting effect and complexity of the model were assessed by the coefficient of determination (*R*^2^), AICc, and root mean square error (RMSE).

### Projections and recommendations for TB elimination

WHO has developed the End TB Strategy milestones and targets, which set out a long-term vision for the global initial elimination of TB by 2035 (Rate < 10/100,000) and the complete elimination of TB by 2050 (Rate < 1/100,000) [6, 12]. The average annual decline rate in last decade was used to predict the future trend of TB and determine whether the milestones and targets can be met. Then, the estimated annual decline rate to meet the TB elimination goal was calculated. To reach the End TB Strategy milestones and targets, the tailored prevention and control suggestions for local was provided by understanding epidemiological characteristics and risk factors obtained by GTWR. According to the epidemic status of TB in Hainan Province, the regions were divided into high, middle and low levels, and corresponding tailored measures were obtained to promote the process of TB elimination.

Data collation was performed using Excel 2019 software, data analysis was performed using R 4.0.1 (R Foundation for Statistical Computing, Vienna, Austria), ArcGIS10.6 software (Esri, Redlands, CA, USA) and the GTWR plugin. This study adopted a two-sided test with a test level of α = 0.05.

## Results

### Epidemiological characteristics of TB

Between 2013 and 2022, there were 64,042 notified cases of TB in Hainan Province, of which 21,876 were bacteriologically positive. The average notification rate of TB was 62.36/100,000. Compared with the same period in 2013, the notification number of TB in 2022 decreased by 37.52%, and the number of bacteriologically positive patients decreased by 19.63%.

#### Temporal distribution

From 2013 to 2022, the notification number and rate of TB in Hainan Province showed a decreasing trend year by year (Table 1).

**Table 1:** TB notification in Hainan Province from 2013 to 2022

Year	Population (100,000)	Case		Notification rate (1/100,000)		Change from the previous year (%)	
		Clinically confirmed	Positive etiology	Clinically confirmed	Positive etiology	Case	Notification rate
2013	895.28	7462	2562	83.35	28.62	-	-
2014	903.48	6973	1703	77.18	18.85	− 6.55	− 7.40
2015	910.82	6853	1837	75.24	20.17	− 1.72	− 2.51
2016	917.13	6776	1712	73.88	18.67	− 1.12	− 1.80
2017	925.76	6672	2025	72.07	21.87	− 1.53	− 2.45
2018	934.31	6432	2142	68.84	22.93	− 3.60	− 4.48
2019	944.72	6277	2573	66.44	27.24	− 2.41	− 3.49
2020	1012.34	6104	2754	60.30	27.20	− 2.76	− 9.25
2021	1020.46	5831	2509	57.14	24.59	− 4.47	− 5.23
2022	1020.02	4662	2059	45.70	20.19	− 20.05	− 20.01

- This study only included data from 2013 to 2022, and it was not possible to calculate the change in 2013 compared to 2012

The EAPC of TB notification rate in Hainan Province from 2013 to 2020 was − 6.88% [95% confidence interval (*CI*): − 5.30%, − 3.69%]. The EAPC of TB notification rates for different age and gender groups was shown in Additional file 1: Figure S1, showing that TB notification rates decreased year by year in most age and gender groups. The Joinpoint model showed that the TB notification rate in Hainan Province showed a significant decline in 2014 (APC = − 7.13, *P* < 0.05), followed by a stable downward trend, and another significant decline in 2022 (APC = − 19.86, *P* < 0.05), as shown in Additional file 1: Figure S2.

**Fig. 1:**
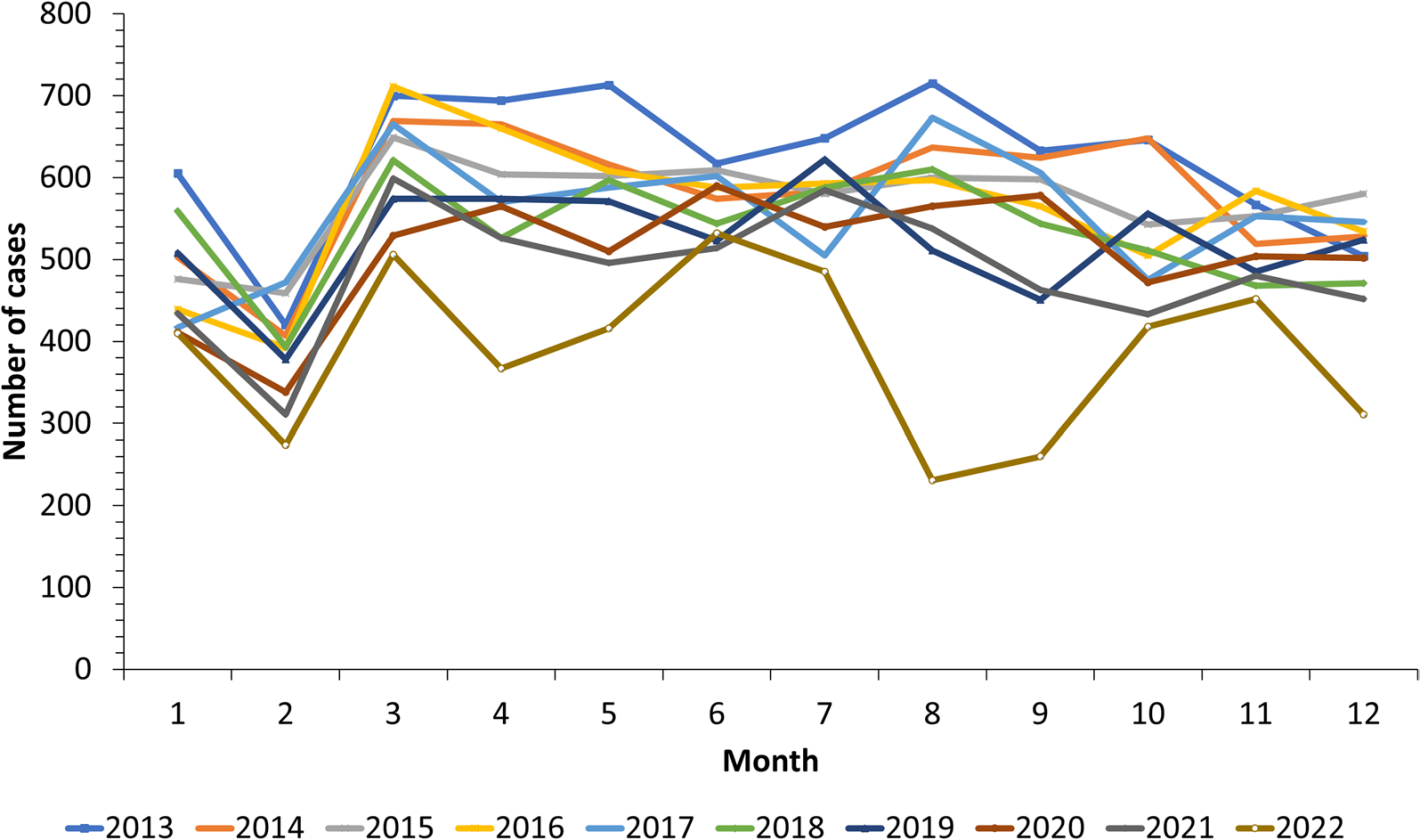
Monthly notification number of TB in Hainan Province from 2013 to 2022

From 2013 to 2022, the average monthly notified cases of TB in Hainan Province were 534 (Standard deviation = 97). There was a clear trough in February, a peak in March, followed by fluctuations in the number of cases, as shown in Fig. 1. Compared with the previous years (2013–2021), the pattern of cases in 2022 was slightly different: In addition to February, there were two troughs occurred in April and August–September. The notification number of TB in Hainan Province was decreasing year by year (Fig. 1). Monthly notification numbers and rates for different cities and counties were shown in Additional file 1: Figure S3, with no clear seasonality, but there may be more notifications in spring.

#### Regional distribution

From 2013 to 2022, TB notification rates in various cities and counties of Hainan Province showed a gradually decreasing trend, as shown in Table 2. High notification areas were mainly located in the central and southern parts of Hainan Province (Lingshui County, Ledong County, Dongfang City, Baoting County, Qiongzhong County, Wanning City, Wuzhishan City). Over time, the range of high notification rate areas had gradually reduced. The global autocorrelation analysis showed that the TB notification rate in Hainan Province was spatially positively correlated from 2016 to 2022. The local autocorrelation analysis showed a significant spatial correlation in the TB notification rate in Hainan Province, with the “high–high” agglomeration area appearing in the central and southern parts of Hainan Province. See Additional file 1: Table S1–S2.

**Table 2:** Tuberculosis notification rate of different regions in Hainan Province from 2013 to 2022

Regions (City and county)	Tuberculosis notification rate (per 100,000 population)									
	2013	2014	2015	2016	2017	2018	2019	2020	2021	2022
Baisha	102.06	70.51	70.18	67.60	71.88	72.09	87.26	87.20	66.95	63.91
Baoting	112.46	128.34	97.20	115.38	93.48	100.13	108.09	97.31	86.46	72.47
Changjiang	90.07	90.91	75.92	75.02	77.16	80.09	86.40	71.83	64.08	45.28
Chengmai	88.11	90.23	90.72	76.58	74.98	61.49	63.61	59.62	53.05	52.06
Danzhou	57.65	53.73	49.20	57.39	57.18	58.99	49.83	52.87	47.83	41.54
Dingan	100.45	86.24	68.29	66.92	78.70	74.93	69.78	61.91	77.80	54.53
Dongfang	97.47	125.48	151.15	155.90	115.20	108.45	68.13	88.00	63.14	48.91
Haikou	49.28	43.94	45.61	47.33	41.59	38.35	43.99	37.62	36.55	28.47
Ledong	143.50	135.34	132.98	120.50	143.81	143.15	121.08	134.62	108.03	95.25
Lingao	57.54	40.72	34.09	66.43	81.57	74.50	64.69	58.04	55.37	42.12
Lingshui	154.84	132.47	143.08	150.32	147.81	161.73	138.03	77.17	97.87	73.94
Qionghai	64.23	47.91	57.71	53.55	51.68	44.41	45.07	47.13	47.33	38.16
Qiongzhong	102.62	80.64	111.74	111.42	86.08	85.46	96.59	95.40	107.62	93.27
Sanya	93.03	71.84	71.57	78.35	77.34	72.49	84.60	58.71	63.63	53.59
Tunchang	107.99	80.50	68.66	69.08	62.24	60.71	44.94	67.58	37.20	32.11
Wanning	147.30	154.92	124.49	69.62	72.35	56.52	54.36	61.90	71.61	42.50
Wenchang	74.18	77.54	74.43	65.14	72.58	68.55	68.15	65.57	53.95	39.49
Wuzhishan	71.43	89.44	71.02	85.85	74.18	103.64	133.88	95.20	95.45	74.93

The numerator of TB notification rate was the number of clinically confirmed cases, which was also the data reported from the Chinese National Disease Control Information System Tuberculosis Surveillance System. No case was recorded in Sansha City

#### Population distribution

**Fig. 2:**
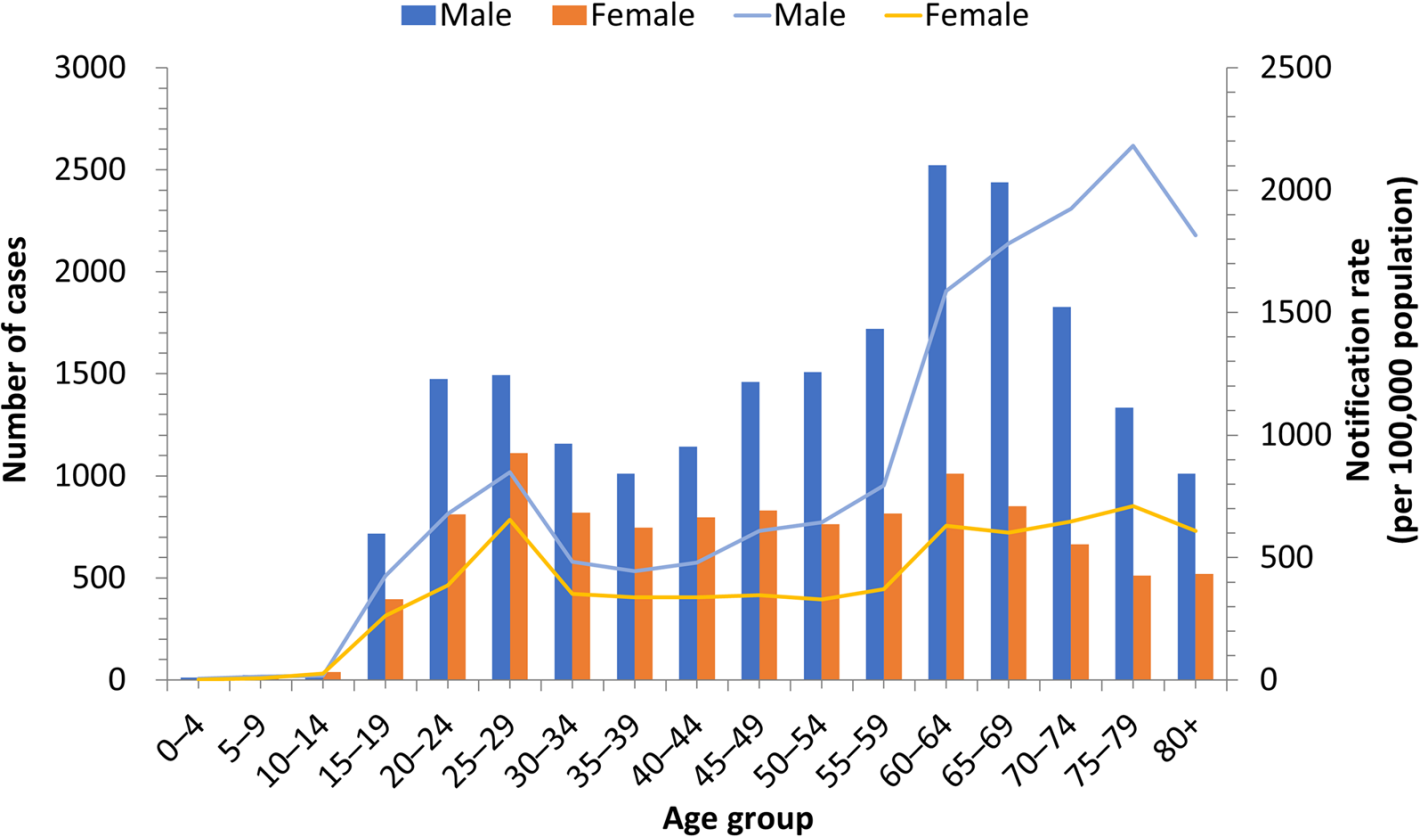
Number and tuberculosis notification rate by gender and age group in Hainan Province from 2013 to 2022

Figure 2 showed the cumulative notification number of TB in Hainan Province from 2013 to 2022 by gender and age group. The age group with the highest notification number was the 60–64 age group. The notification rate for the 0–14 age group was very low. After 14 years old, the notification rate gradually increased, reaching a small peak in the 20–24 age group, then fluctuated upwards with age, reaching the highest value in the 75–79 age group, and then gradually decreased. The notification number and rate of males were higher than females in all age groups. The notification rate of each age group decreased over time, as shown in Additional file 1: Figure S4.

Male TB patients heavily outnumbered females, accounting for 76.33% of the cumulative number of cases over ten years. Farmers, homemakers and the unemployed, retirees, students, and workers were the top five populations, accounting for 88.09% of all cases. Farmers were the most common group, with a total of 43,422 cases notified in ten years (67.80%). Han nationality and Li minority were the main ethnic groups in cases, accounting for 98.89% of the cumulative number of cases over ten years (Additional file 1: Table S3).

### Migrant features of TB cases

Cases within county of the district, between districts and counties within the city, between cities in Hainan Province, and outside Hainan Province accounted for 94.09%, 0.60%, 1.82% and 3.49%, respectively. Most of TB cases in each city or county in Hainan Province were in their own counties, of which Sanya City accounted for a relatively small proportion (68.94%). There were very few migrant cases among different districts in the same city, cases of other cities in the province were mainly flowing to Sanya City and Haikou City.

**Fig. 3:**
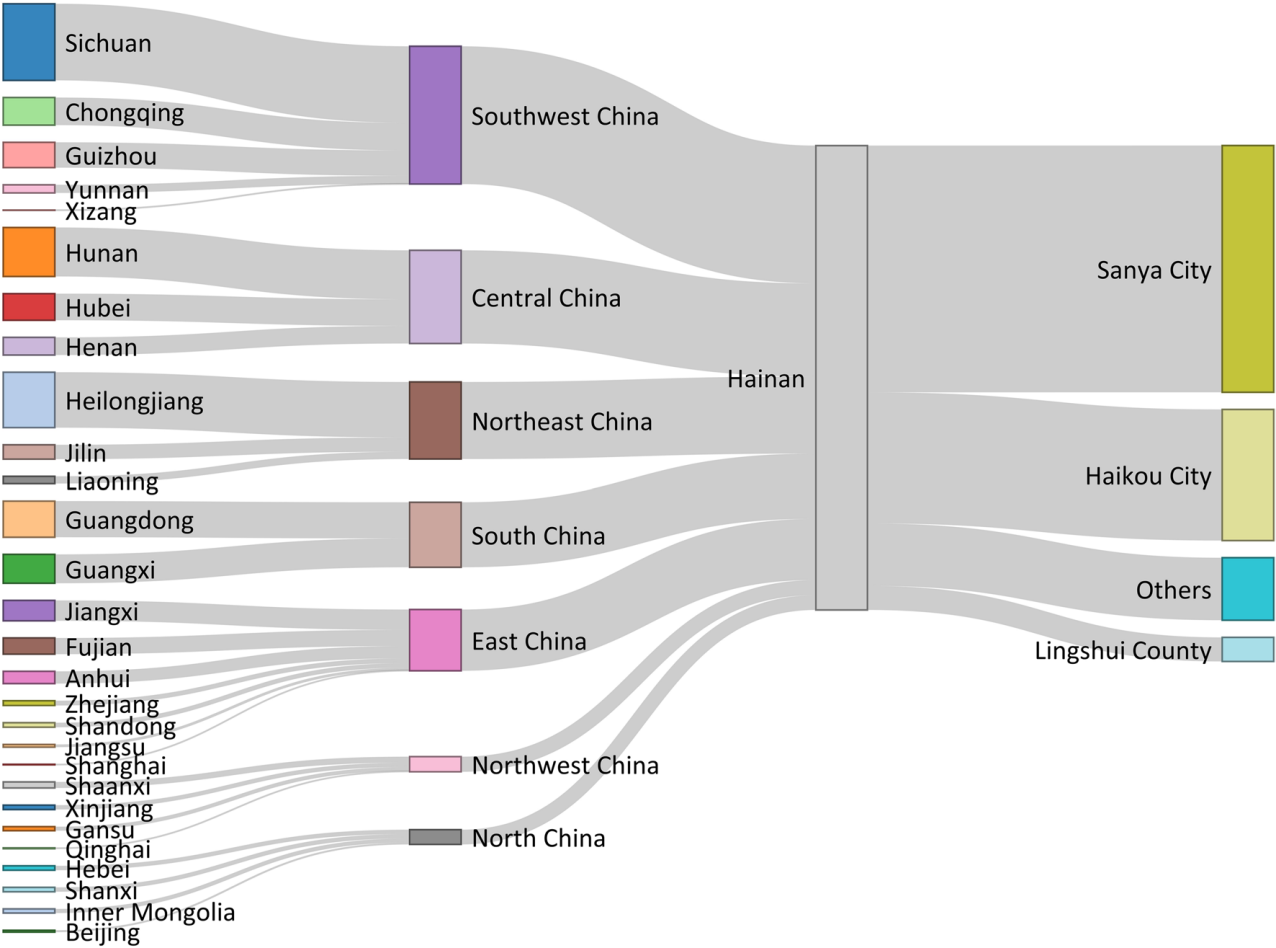
Provinces of origin of tuberculosis cases from outside Hainan Province (The first layer was the provincial-level administrative division, and the second layer was the geographic region division.)

There were 2236 cases from outside Hainan Province, these cases mainly came from Sichuan Province (369 cases), Heilongjiang Province (267 cases), Hunan Province (236 cases), Guangdong Province (174 cases), and Guangxi Zhuang Autonomous Region (139 cases), accounting for 53% of migrant cases, as shown in Fig. 3. Cases from other provincial-level administrative divisions (PLADs) mainly flowed into Sanya City (53.18%), Haikou City (28.18%) and Lingshui County (5.19%), and cases from other PLADs in Sanya City accounted for 19.81% of total TB cases.

There was no significant peak in the number of migrant treatments each month, with relatively fewer cases in January and February. The gender ratio of migrant cases was 3.01:1, with males heavily outnumbering females. Among the different age groups, the number of migrant cases was highest among the 45–49 age group. Farmers and migrant workers, as well as homemakers and the unemployed, accounted for a larger proportion (62%). Details were showed in Additional file 1: Figure S5–S7.

### Finding patterns of TB cases

From 2013 to 2022, among the TB cases in Hainan Province, 63,291 patients (98.83%) were notified through passive-case finding approaches, 512 patients (0.80%) through active-case finding approaches and 239 patients (0.37%) were found in other ways (Table 3). Among TB patients, there were 63,831 cases (99.67%) of active pulmonary TB. The number of bacteriologically negative patients was 40,888 (63.85%), and the number of bacteriologically positive patients was 21,810 (34.06%). Details were showed in Additional file 1: Figure S8.

**Table 3:** Proportion of tuberculosis patients from different sources in Hainan Province from 2013 to 2022

Methods of discovery	Case (%)
Passive case finding	
Symptom based medical consultation	21,955 (34.28)
Referral	32,047 (50.04)
Tracing	6753 (10.55)
Recommendations by primary health centers	2536 (3.96)
Active case finding	
Physical examination	476 (0.74)
Active screening	36 (0.06)
Other ways	239 (0.37)
Total	64,042

The median of patient delay days was 30 [interquartile range (IQR): 10–61], and there were no significant gender differences in the duration of patient delay. The median duration of patient delay in students was lower than that in other occupational groups. The low age group (≤ 24) had a shorter duration of patient delay of 24 (IQR: 6–46) days than other age groups. The median duration of patient delay of Han population 29 (IQR: 8–55) days was lower than that of Li population 32 (IQR: 15–65) days and other populations 33 (IQR: 14–73.75) days. Details were provided in Additional file 1: Table S4.

### Regression analysis between TB notification rate and socio-economic status

The spatial and temporal distributions of the four influencing factors were shown in Additional file 1: Table S5. The result from GTWR model showed that Adjusted *R*^2^ was 0.85, AICc was 1514.32, RMSE was 11.46, indicating that the model had a high goodness of fit (Additional file 1: Table S6). The regression model was tested to be free of multicollinearity problems.

The temporal and spatial variation of the estimated values of each parameter of the GTWR model was shown in Table 4. This study found that the coefficient estimates of socioeconomic indicators varied with the change in spatio-temporal position. The estimated coefficient values were statistically significant by statistical test (*P* < 0.05). The results of GTWR model showed that the parameter values of proportion of rural population in the southern and southeastern areas of Hainan Province were positive (IQR: 0.03–5.20). The higher rural population proportion, the higher local TB notification rate. For instance, Qiongzhong and Wanning counties with higher notification rates had higher proportion of rural population. The lower GDP per capita, the higher TB notification rate (IQR: − 4.39 to − 0.70). For example, the GDP per capita in Ledong county, Baoting County and Wuzhishan City were at a lower level and the TB notification rates were higher. The numbers of medical institutions and health personnel per 10,000 people were lower in the southeast and southwest areas of Hainan Province (Lingshui County, Baoting County, Dongfang City, Wanning City), and the estimated values of these areas were negative (IQR: − 20.77 to − 0.04), that is, the lower numbers of medical institutions and health personnel per 10,000 people, the higher TB notification rate.

**Table 4:** Coefficient estimates of influencing factors for tuberculosis in Hainan Province from 2013 to 2022

Regions	Proportion of rural population-coefficient estimates			GDP per capita-coefficient estimates (10^−3^)			Number of medical institutions per 10,000 people-coefficient estimates			Number of health personnel per 10,000 people-coefficient estimates		
	P_25_	P_50_	P_75_	P_25_	P_50_	P_75_	P_25_	P_50_	P_75_	P_25_	P_50_	P_75_
Baisha	− 4.84	− 2.24	− 0.39	− 4.39	− 2.41	− 1.91	− 8.00	− 2.11	3.05	− 2.52	− 0.67	0.39
Baoting	− 0.20	− 0.04	1.16	− 1.80	− 1.24	− 0.70	− 18.01	− 10.94	− 6.65	0.08	0.31	1.02
Changjiang	− 4.78	− 1.70	− 0.28	− 4.15	− 2.14	− 2.05	− 9.32	− 6.65	1.40	− 2.52	− 1.56	0.27
Chengmai	− 0.26	0.60	0.67	− 0.14	0.28	0.37	− 0.20	1.12	11.91	− 0.12	− 0.10	− 0.04
Danzhou	− 0.74	− 0.02	0.00	− 0.45	− 0.10	0.30	1.72	9.72	16.36	− 1.14	− 0.67	0.13
Dingan	− 1.33	− 0.70	0.26	− 0.43	− 0.31	0.11	2.18	5.56	9.77	− 0.52	− 0.40	− 0.20
Dongfang	− 3.89	− 0.60	− 0.14	− 3.29	− 2.19	− 1.87	− 9.59	− 7.06	− 1.66	− 2.04	− 1.26	0.29
Haikou	− 0.86	0.10	0.36	− 0.17	− 0.11	− 0.06	0.90	2.27	9.77	− 0.38	− 0.24	− 0.14
Ledong	− 0.98	− 0.08	0.22	− 1.94	− 1.60	− 1.04	− 9.28	− 4.14	− 0.58	− 1.17	− 0.10	0.37
Lingao	− 0.64	0.47	0.54	− 0.10	0.15	0.30	0.87	3.10	10.73	− 0.28	− 0.12	− 0.09
Lingshui	− 0.32	− 0.20	0.26	− 2.04	− 1.76	− 1.28	− 20.77	− 13.38	− 7.32	− 0.33	0.38	0.80
Qionghai	− 1.74	− 0.48	0.12	− 1.25	− 0.97	− 0.09	2.60	3.49	17.44	− 0.21	− 0.17	− 0.15
Qiongzhong	0.03	0.18	0.89	− 0.58	0.29	1.63	− 4.73	− 4.17	− 1.97	0.13	0.37	0.59
Sanya	− 0.06	0.46	1.14	− 1.33	− 0.96	− 0.25	− 9.30	− 6.77	− 4.70	− 0.30	0.24	0.30
Tunchang	0.58	0.75	1.03	− 0.33	− 0.06	0.01	7.69	8.25	10.39	0.17	0.34	0.45
Wanning	0.22	0.61	5.20	− 0.42	0.36	0.98	− 11.59	− 0.10	6.25	− 2.97	− 1.66	− 0.86
Wenchang	− 0.98	− 0.05	0.18	− 0.72	− 0.47	− 0.30	1.20	1.43	8.77	− 0.41	− 0.17	− 0.13
Wuzhishan	− 1.76	− 0.34	− 0.32	− 2.29	− 2.11	− 1.31	− 12.84	− 7.04	− 5.47	− 0.80	0.07	0.45

No case was recorded in Sansha City

### TB elimination in Hainan Province

**Fig. 4:**
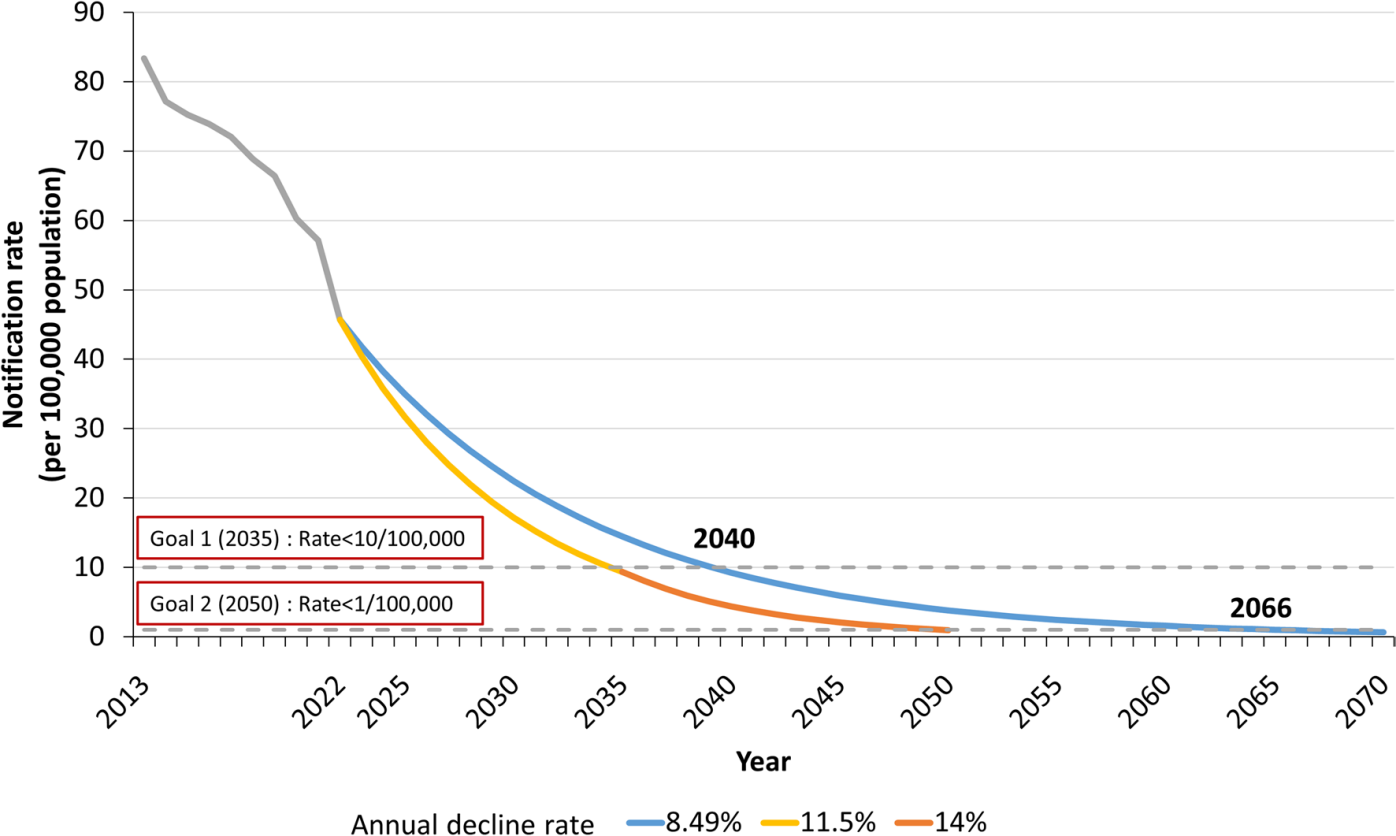
Tuberculosis notification rate in Hainan Province last decade and future prediction (Blue was the average annual decline rate in last decade, yellow and orange were the estimated annual decline rates to meet the TB elimination goal.)

Taking the average annual decline rate (8.49%) of TB notification rate last decade in Hainan Province as a reference, the notification rate would be less than 10/100,000 in 2040 and less than 1/100,000 in 2066. To achieve the goals of TB end in 2050 set by WHO, the TB notification rate in Hainan Province would need to maintain an average annual decline rate of 11.5% in the future, so as to reduce the notification rate to less than 10/100,000 in 2035, and then further would increase the average annual decline rate to 14%, and complete the TB notification rate less than 1/100,000 in 2050, as shown in Fig. 4.

Based on the TB notification rate and epidemiological characteristics of each city and county in Hainan Province, they were divided into three groups: high, medium and low, as shown in Table 5. Corresponding projections of the TB notification rate in the three groups were shown in Additional file 1: Figures S9–S11, none of the three groups would achieve TB elimination on current trends. Further TB prevention and control measures are needed to increase the annual decline rate, especially in regions with high TB notification rate. The annual decline rates of high, medium and low groups would be 15%, 12% and 11.5%, respectively, before 2035 to achieve TB elimination targets. According to the characteristics of each region, prevention and control measures should be taken according to local conditions to end TB, as shown in Table 5. For example, although the TB notification rates in Haikou and Sanya were low, the proportion of migrant cases was relatively high (based on the results of Migrant features of TB cases), and the effect of number of medical institutions per 10,000 people was negative but the number was small (based on the results of GTWR). Therefore, it is suggested that more attention should be paid to the prevalence of TB in the migrant population and more TB specialized hospitals should be set up in these areas. In the same way, for regions with high TB notification rates, such as Lingshui and Ledong, corresponding policy recommendations were obtained based on the local TB prevalence and GTWR results (Table 4). In addition, because of the complex situation in areas with high TB notification rates, measures for further subdivision of these areas were provided in Additional file 1: Table S7 for details.

**Table 5:** Suggestions on prevention and control of tuberculosis in Hainan Province

Group (Based on average annual TB notification rate)	Regions	TB control	Evidences from results in this study
Low (< 60/100,000)	Haikou City, Sanya City	More TB specialized hospitals, TB surveillance in migrant population	*“Migrant features of TB cases”, “Regression analysis between TB notification rate and socio-economic status”*
Medium (< 80/100,000)	Qionghai City, Wenchang City, Chengmai County and other regions	More TB specialized hospitals and health personnel	*“Regression analysis between TB notification rate and socio-economic status”*
High (≥ 80/100,000)	Lingshui County, Ledong County, Dongfang City, Baoting County, Qiongzhong County, Wanning City, Wuzhishan City	More attention on rural and poor population, More TB specialized hospitals and health personnel	*“Regression analysis between TB notification rate and socio-economic status”*

## Discussion

Through understanding the epidemiological characteristics and risk factors of TB last decade in Hainan Province, this study enhanced the knowledge on current epidemic of TB in Hainan Province to provide scientific suggestions and references for local TB elimination work.

### TB notification rate varied with the time and space correlated with migrant and minority population

The epidemiological study analyzed the spatial–temporal distribution of TB in Hainan Province from 2013 to 2022, and showed following four feature. Firstly, with the implementation of the TB control programme since 2014, the TB notification rate in Hainan Province has decreased significantly and maintained a stable downward trend. The supported by the fact that a total of 64,042 TB cases were notified in Hainan Province from 2013 to 2022, the number of TB cases and pathogen-positive cases notified in 2022 decreased by 37.52% and 19.63% compared with that in 2013, respectively. However, the average annual notification rate of TB in Hainan Province was 62.36/100,000, which was higher than the national average level, so that more harder efforts are necessary to mitigate the relatively high risk of TB facing in Hainan Province [9]. This decline trend of TB transmission could potentially be attributed to the adoption of the "integrated" TB control framework in China, wherein specialized TB hospitals assumed the roles of diagnosing, treating, and overseeing TB cases. This integrated approach has markedly enhanced the efficiency of TB prevention and management, and TB control programme in China and Hainan has gradually improved [6, 25].

Secondly, the TB notification rate was higher in some regions of Hainan Province, particularly among males, old people, and farmers. The prevalence of TB in Hainan Province showed no significant seasonality presented in the spatial–temporal distribution study. However, the TB notification rate was relatively higher during spring, possibly due to the climate change and environmental variation [7, 8]. The central and southern regions of Hainan Province exhibited higher TB notification rates, which are largely attributed to their higher population density, weak economic conditions, and limited access to medical resources [26, 27]. The results of study by Christian et al. and Knut et al. showed that people contact can increase the spread of TB, and people with low socioeconomic level were more likely to develop TB due to poor living conditions and weak health awareness [26, 27]. The notification rate was higher in people over 60 years old, and the males with a higher proportion surpassed the global and Chinese averages, which likely linked to local male lifestyles, working environments, and personal health management. Particularly unique habits, like more social gatherings, among the males in Hainan Province may increase their susceptibility to TB infection. Additionally, unhealthy habits such as smoking and alcohol addiction can weaken male immunity, elevating the risk of TB [10, 28]. Li et al. also found that men in Hainan Province had the custom of gathering to drink tea and chat, which aggravated the spread of TB [10]. Moreover, the males may show lower health awareness and engage in fewer regular check-ups [29]. The proportion of farmers among TB patients was also relatively high, possibly due to the poor living conditions in rural areas [26].

Thirdly, the unique characteristics of migrate population frequently traveling to Hainan Province have become the bridge for spreading TB transmission. It supported by fact that imported cases mainly originated from Sichuan Province, Heilongjiang Province, Hunan Province, Guangdong Province, and Guangxi Zhuang Autonomous Region, which was in consistence with the provinces where majority of migrant came from [30] as well as the high TB notification rate observed in these regions [16, 23]. The number of migrant cases was relatively low in January and February, possibly due to the Chinese New Year, during which many migrant workers may return home to reunite with their families [9]. The study by He et al. showed an association between cross-domain population migration and TB incidence [16]. Firstly, population migration increases the contact between people, and the risk of infectious diseases such as TB increases greatly. Secondly, population migration in China is often from areas with a low level of economic development to the high, and people in areas with a low level of economic development are more likely to carry *Mycobacterium tuberculosis* [16, 31]. TB primarily spread through airborne transmission, and the interprovincial mobility of these cases could potentially facilitate TB transmission between regions, posing a hidden risk of TB outbreaks in Hainan Province [16, 31].

Fourthly, TB patients in Hainan Province were mainly found passively, with low positive detection rate and delay in seeking medical care. It is noticed that bacteriologically negative patients were more than positive patients, which still possess a certain degree of infectivity and may have a certain impact on TB prevalence in the community [32]. The median duration of patient delay in seeking medical attention for TB patients in Hainan Province was 30 (10, 61) days, higher than the national average [33]. Among different occupational groups, students had lower duration of patient delay, likely because they have easier access to relevant health education and medical resources, enabling them to seek diagnosis and treatment earlier [34]. Additionally, the delay in seeking medical attention was lower among the Han ethnic group compared to minority ethnic groups like the Li people, possibly due to cultural differences between different ethnic groups, unequal distribution of medical resources, and varying levels of awareness of TB [35, 36].

### Correlation between TB notification rate and socio-economic status

The investigation employed with GTWR model showed that the impact factors of TB notification rate in Hainan Province were more likely associated with social and economic indicators. Results showed that per capita GDP (factors reflecting the economic development level) and number of medical institutions and health personnel per 10,000 population (factors reflecting the level of medical and health services) mainly influenced the high TB notification rate in some cities and counties of Hainan Province. Additionally, other risk factors may also exist in some cities and counties. For example, Qiongzhong County and Wanning County should pay attention to the TB prevention and control situation among their rural populations.

Firstly, lower per capita GDP in most areas was associated with higher TB notification rates. TB prevalence is not only a medical issue but also a social issue. Economically developed regions have higher population densities but better natural conditions, more advanced medical facilities, and higher levels of TB prevention and control knowledge, resulting in lower TB notification rates. Conversely, economically undeveloped regions have a lower population density but relatively abundant per capita medical resources. However, the uneven development of regional economy contributes to spatial differences in TB incidence in the study area [37–39], due to limitations imposed by the natural environment, educational level, and customs, TB prevention and control efforts may not yield immediate results in these regions [26]. Previous studies have revealed associations between TB and per capita GDP, poverty, living standards, and malnutrition [26, 27]. Christian et al. and Knut et al. suggested that most risk factors for TB were associated with social conditions. People from lower socioeconomic groups generally have more contact with people with active disease, are more likely to live and work in crowded environments, have lower levels of health awareness, and have less access to quality health care than people from higher socioeconomic groups [26, 27]. A European study showed that disparities in TB prevalence were due to uneven economic development. There was a significant negative correlation between GDP and TB incidence [40, 41].

Secondly, the higher the number of medical institutions and health personnel in the central and some developed cities of Hainan Province, the lower the TB notification rate. The number of medical and health institutions and health personnel can reflect the level of medical security and the treatment capacity of the medical system to some extent. The greater the number of medical and health institutions and health personnel, the higher the level of health services can be used to detect TB earlier and reduce its transmission [6, 40, 42]. Both studies by Zhang and Wang et al*.* showed a close relationship between the level of medical resources and TB notification rate. Insufficient medical resources can not screen more potential TB patients, and the prevention and control of TB patients is also weak, resulting in the transmission of TB can not be effectively interrupted [40, 42]. The results of GTWR provided a possible scientific reference for regional TB prevention and control efforts, contributing to the improvement and achieving the goal of precise prevention and control measures.

### Projection patterns for TB elimination in Hainan Province

This study also predicted the future prevalence of TB in Hainan Province, and the results showed that the 2035 (Rate < 10/100,000) and 2050 (Rate < 1/100,000) targets set by WHO would not be reached if the current intervention strategy is not changed with the same downward trend of TB notification rate. Therefore, the prevention and control of TB in Hainan Province needs to be further strengthened in the future, insisting on highlighting key points, adapting measures to local conditions and guiding according to classification. The Chinese government has further issued the Guiding Tuberculosis Control Through the Healthy China Initiative 2019–2030 to guide local prevention and control work [43].

Based on the above research results, first of all, the following strengthening measures for TB elimination in whole Hainan Province were proposed: (1) TB screening in key populations, (2) prevention and control of TB in migrant population, (3) active surveillance. Firstly, it is recommended that local TB screening be strengthened in key populations, expand screening coverage for key groups such as males, people over 60 years old, and rural populations. Secondly, it is necessary to promote the prevention and control of TB in migrant population, strengthen inter-departmental cooperation, improve the working and living conditions of migrant population in densely packed places, strengthen environmental health improvement, and carry out symptom screening. Thirdly, active surveillance should be strengthened, and the designated TB medical institutions, disease control institutions and primary medical and health institutions should strengthen cooperation, and timely TB examination should be carried out for the symptomatic patients. The application of new diagnostic techniques is promoted to improve the positive detection rate of pathogens in TB patients.

Secondly, the annual decline rates of high, medium and low groups would be 15%, 12% and 11.5%, respectively, before 2035 to achieve TB elimination targets. It is necessary to further analyze the characteristics of local epidemic and carry out tailored and precise prevention to reduce the risk of TB in Hainan Province. (1) Regions with low TB notification rate need more TB specialized hospitals to provide more adequate medical services, and more attention should be paid to the TB epidemic of migrant population outside the province and in other cities in the province. Since the establishment of free trade port in Hainan Province, cross-border population migration has increased. It is necessary to establish and improve the epidemic information reporting system. Health education activities on TB prevention and control were held regularly in the gathering places of migrant population, and environmental sanitation was improved. (2) The regions with medium TB notification rate, like Qionghai City and others, need to improve the level of medical and health services, increase TB specialized hospitals and health personnel. (3) In regions with high TB notification rate, more attention should be paid to TB prevention and control in rural and poor population, and more TB specialized hospitals and health personnel should be provided to improve the level of medical services. Comprehensive universal TB census and centralized isolation and treatment of patients with infectious disease should be carried out to promote the rapid decline of TB cases. Focusing on TB prevention and control in these regions will help Hainan Province achieve TB elimination.

In addition, combined with the Global Plan to End TB (2023–2030) [44], Hainan can also strengthen TB screening in students, diabetes patients, people living with HIV/AIDS. To curb the further development of drug-resistant TB, improve the laboratory diagnostic capacity of drug-resistant TB, and promote the standardized diagnosis and treatment of drug-resistant TB. Scientific research on TB should be intensified, new vaccines should be developed, and the protective efficacy of vaccines should be improved.

Furthermore, the COVID-19 pandemic had a continued negative impact on the accessibility of TB diagnosis and treatment services from 2020 to 2022, as well as the burden of TB [1, 2]. The results of this study indicated a considerable reduction in the TB notification rate starting from 2020. This decline could be attributed to an increased number of undiagnosed and untreated TB patients as a consequence of the pandemic's impact. With the outbreak of COVID-19, diagnostic services were disrupted due to TB staff supporting the prevention and control of the COVID-19 epidemic, resulting in insufficient TB services and an overloaded healthcare system. Consequently, laboratories might have struggled to diagnose TB and other diseases in a timely manner. Moreover, COVID-19 related isolation and lockdown measures have made it more difficult for TB patients to seek medical attention promptly, leading to delays in diagnosis and treatment [45, 46].

This study had certain limitations. Firstly, Hainan Province is a tourist attraction in China with high population mobility, which increases the difficulty of TB prevention. The study only obtained rudimentary information about migrant population without details on residence time and the size of migrant population, which may result in relatively biased analyses of related factors. Secondly, TB may also be influenced by individual lifestyle habits, regional customs, and other potential factors. These factors were not comprehensively controlled in this study, potentially leading to biased results. Third, there was a lack of field studies on specific populations to obtain the evaluation of the effect of the current measures. To gain a more comprehensive understanding of TB transmission and prevention, future research may consider further analysis of the impact of migrant populations on TB transmission and corresponding field studies to understand their individual living habits, regional customs, and existing TB prevention, evaluate current measures, and improve TB control through interventions.

## Conclusions

The TB notification rate in central and southern Hainan Province is high but has been decreasing recently, indicating progress towards TB elimination. It is important to focus on TB prevalence in males and farmers and take tailored measures for elimination. Strengthening TB surveillance and intervention on migrant population from other provinces like Sichuan, Heilongjiang, and Hunan can help reduce the spread of TB. Active surveillance should be strengthened to improve the diagnosis and treatment of TB. Improving socio-economic conditions in areas with lower development and poor healthcare is crucial for TB elimination. Proper allocation of medical resources and tailored measures in each district will ensure effective implementation of TB elimination goals.

### Supplementary Information

**Additional file 1: Figure S1.** EAPC of TB notification rate by age and gender in Hainan Province from 2013 to 2022. **Figure S2**. Annual trend of TB notification rate in Hainan Province from 2013 to 2022. **Figure S3**. Monthly notification number and rate of TB in different cities and counties in Hainan Province from 2013 to 2022. **Table S1**. *Moran's I* index results of notification rate of TB at the city/county level in Hainan. **Table S2**. LISA of TB notification rate in Hainan Province. **Figure S4**. Changes in the notification rate of TB with age in Hainan Province from 2013 to 2022. **Table S3**. TB cases with different population characteristics in Hainan Province from 2013 to 2022. **Figure S5**. Time distribution of TB cases from outside Hainan Province. **Figure S6**. Age and gender distribution of migrant TB cases outside Hainan Province. **Figure S7**. Occupational distribution of migrant TB cases outside Hainan Province. **Figure S8**. Notification distribution of TB patients in Hainan Province from 2013 to 2022. **Table S4**. Patient delay situation of TB patients in Hainan Province. **Table S5**. Influencing factors for TB in Hainan Province from 2013 to 2022. **Table S6**. Results from GTWR model analysis. **Figure S9**. TB notification rate in regions with low TB notification rate last decade and future prediction. **Figure S10**. TB notification rate in regions with medium TB notification rate last decade and future prediction. **Figure S11**. TB notification rate in regions with high TB notification rate last decade and future prediction. **Table S7**. Main risk factors influencing the prevalence of TB in different cities and counties.

## Data Availability

The TB notification data in Hainan Province from 2013 to 2022 were collected from the Chinese National Disease Control Information System Tuberculosis Surveillance System. We would like to share statistical results of this study. The datasets used and/or analysed during the current study are available from the corresponding author on reasonable request.

## Abbreviations

TB: Tuberculosis
EAPC: Annual estimated percentage change
LISA: Local indicator of spatial association
GDP: Gross domestic product
GTWR: Geographically and temporally weighted regression
AICc: Akaike’s information criterion
RMSE: Root mean square error
COVID-19: Coronavirus disease 2019
