# Supplementary material for: Toward tuberculosis elimination by understanding epidemiologic characteristics and risk factors in Hainan Province, China
Source: Infect Dis Poverty. 2024 Feb 27;13:20. doi: 10.1186/s40249-024-01188-2 (PMC10898115; doi:10.1186/s40249-024-01188-2)
Supplement: Supplementary file 1 — Additional file 1: Figure S1. EAPC of TB notification rate by age and gender in Hainan Province from 2013 to 2022. Figure S2. Annual trend of TB notification rate in Hainan Province from 2013 to 2022. Figure S3. Monthly notification number and rate of TB in different cities and counties in Hainan Province from 2013 to 2022. Table S1. Moran's I index results of notification rate of TB at the city/county level in Hainan. Table S2. LISA of TB notification rate in Hainan Province. Figure S4. Changes in the notification rate of TB with age in Hainan Province from 2013 to 2022. Table S3. TB cases with different population characteristics in Hainan Province from 2013 to 2022. Figure S5. Time distribution of TB cases from outside Hainan Province. Figure S6. Age and gender distribution of migrant TB cases outside Hainan Province. Figure S7. Occupational distribution of migrant TB cases outside Hainan Province. Figure S8. Notification distribution of TB patients in Hainan Province from 2013 to 2022. Table S4. Patient delay situation of TB patients in Hainan Province. Table S5. Influencing factors for TB in Hainan Province from 2013 to 2022. Table S6. Results from GTWR model analysis. Figure S9. TB notification rate in regions with low TB notification rate last decade and future prediction. Figure S10. TB notification rate in regions with medium TB notification rate last decade and future prediction. Figure S11. TB notification rate in regions with high TB notification rate last decade and future prediction. Table S7. Main risk factors influencing the prevalence of TB in different cities and counties. [file 40249_2024_1188_MOESM1_ESM.docx]

**Toward tuberculosis elimination by understanding epidemiologic characteristics and risk factors in Hainan Province, China**

Changqiang Zhou^1^, Tao Li^2^, Jian Du^3^, Dapeng Yin^4*^, Xiujun Li^1,5*^, Shixue Li^1*^

**1** Department of Biostatistics, School of Public Health, Cheeloo College of Medicine, Shandong University, Jinan, Shandong, China.

**2** National Center for Tuberculosis Control and Prevention, Chinese Center for Disease Control and Prevention, Beijing, China.

**3** Clinical Center on TB Control, Beijing Chest Hospital, Capital Medical University/Beijing Tuberculosis & Thoracic Tumor Research Institute, Beijing, China.

**4** Hainan Center for Disease Control and Prevention, Haikou, Hainan, China.

**5** Research Center for Tuberculosis Control, Shandong University, Jinan, Shandong, China.

*They contributed equally as co-corresponding authors.

*Correspondence to:

Dapeng Yin,

Email: yindapeng@hainan.gov.cn;

Hainan Center for Disease Control and Prevention, Haikou 570203, China.

Xiujun Li,

Email: [xjli@sdu.edu.cn](mailto:xjli@sdu.edu.cn);

44# Wenhuaxi Road, Lixia District, Jinan, Shandong 250012, People's Republic of China.

Shixue Li,

Email: shixueli@sdu.edu.cn;

44# Wenhuaxi Road, Lixia District, Jinan, Shandong 250012, People's Republic of China.

**Epidemiological characteristics of TB**

***Temporal distribution***


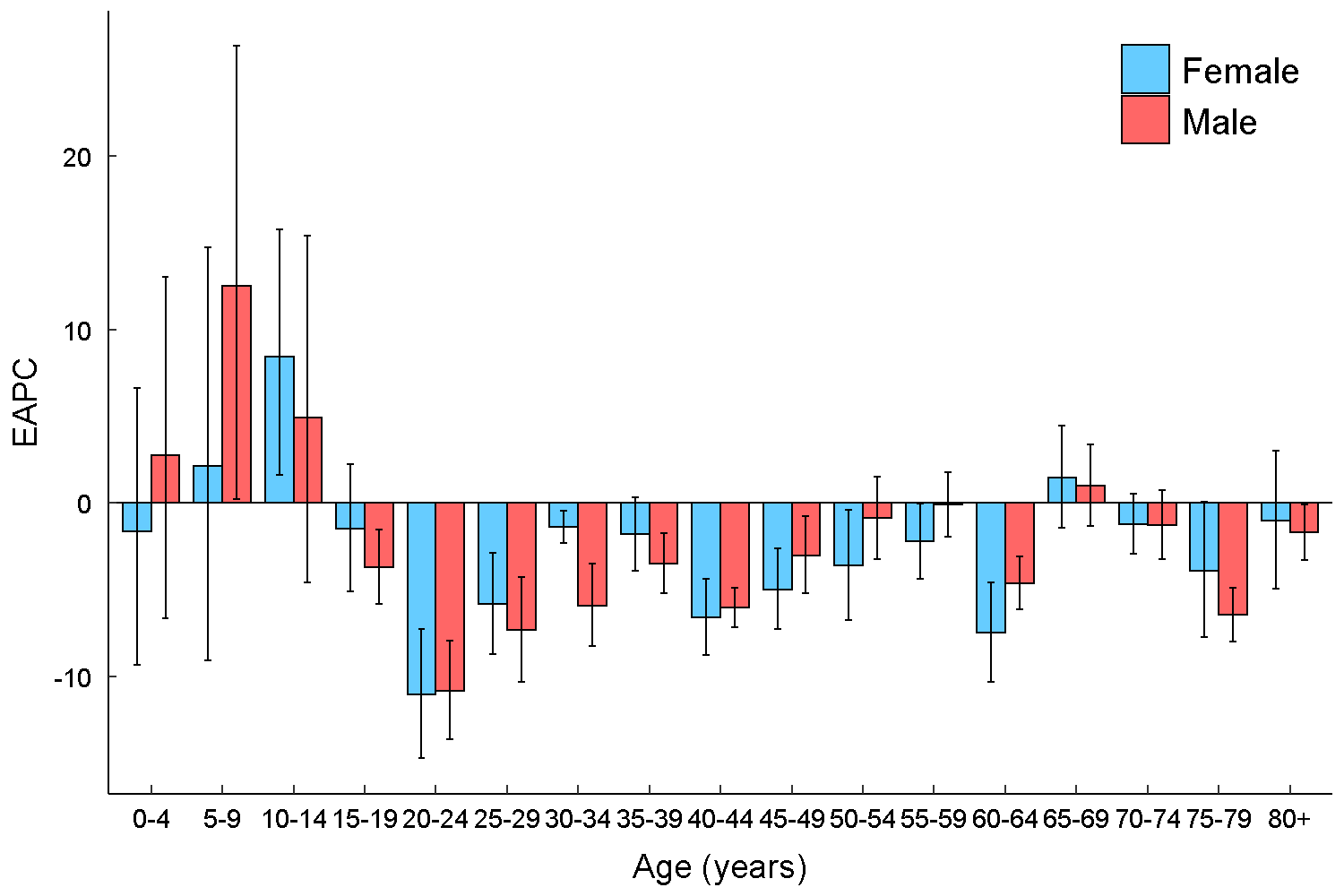


**Figure S1** EAPC of TB notification rate by age and gender in Hainan Province from 2013 to 2022

**Parameter setting of Joinpoint model**: We used 10 years of data and a log-linear model because TB notification rates were not normally distributed. The grid search method (GSM) was used to fit the model. The model selection was based on the Bayesian information criterion (BIC). The minimum number of Joinpoints was set to 0 and the maximum was set to 5, and the final selected model was 3 Joinpoints. The annual percentage change (APC) and average annual percentage change (AAPC) and 95% CI were main outcome indicators in Joinpoint model. The Confidence Intervals of APC and AAPC were calculated using the Empirical Quantile method.


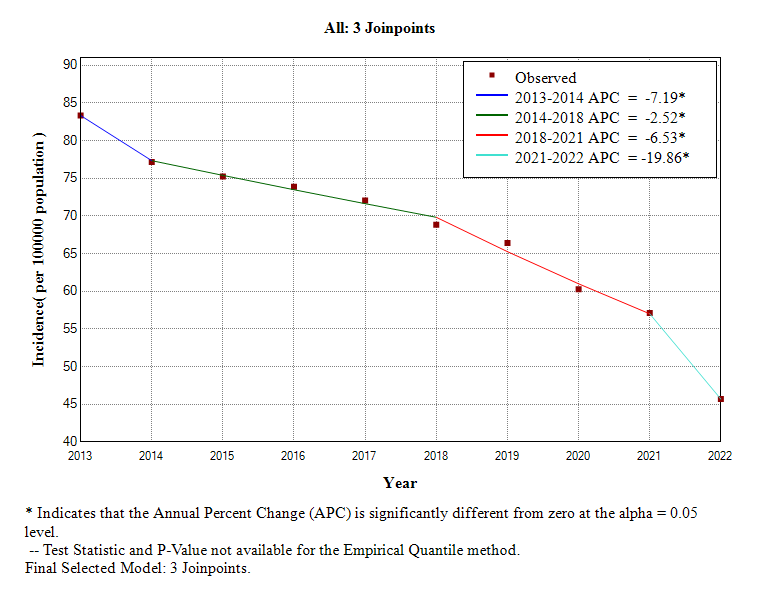


**Figure S2** Annual trend of TB notification rate in Hainan Province from 2013 to 2022


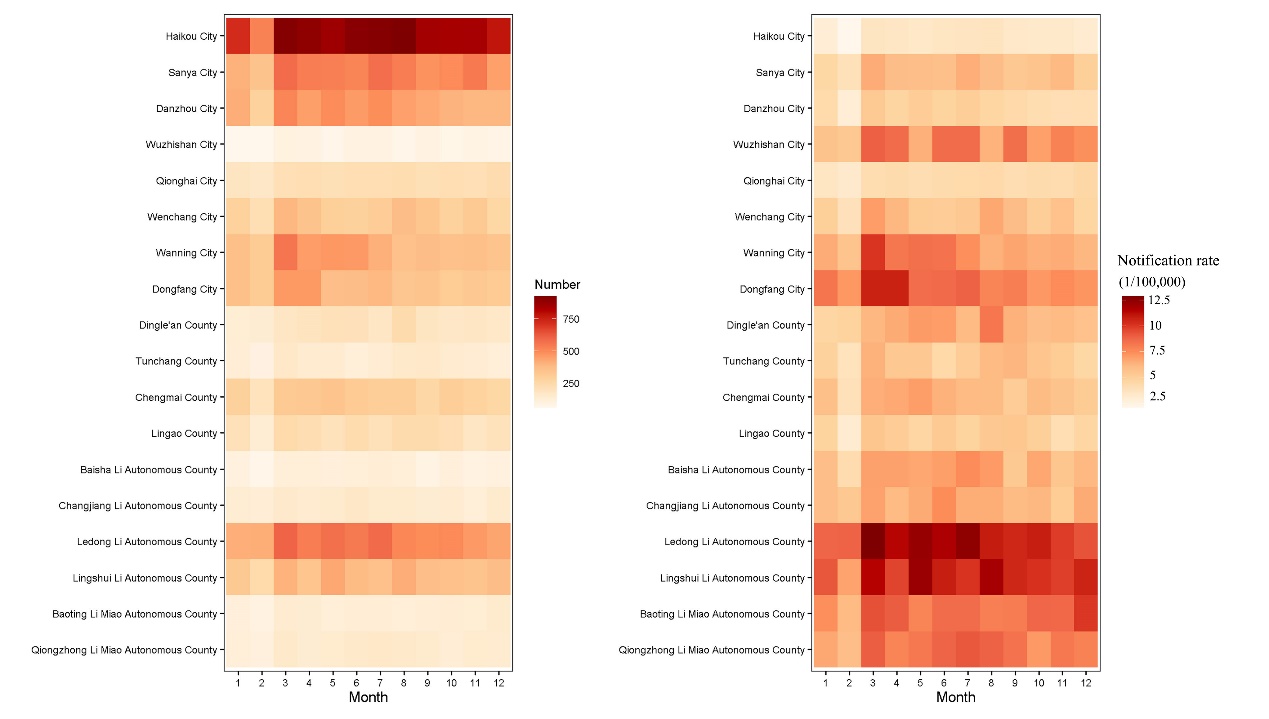


**Figure S3** Monthly notification number and rate of TB in different cities and counties in Hainan Province from 2013 to 2022

***Regional distribution***

**Table S1** Moran's *I* index results of notification rate of TB at the city/county level in Hainan Province

| **Year** | **Moran's *I*** | ***Z-value*** | ***p-value*** |
| --- | --- | --- | --- |
| 2013 | 0.02 | 1.78 | 0.07 |
| 2014 | 0.02 | 1.79 | 0.07 |
| 2015 | 0.02 | 1.79 | 0.07 |
| 2016 | 0.05 | 2.61 | 0.01* |
| 2017 | 0.02 | 1.75 | 0.08 |
| 2018 | 0.05 | 2.49 | 0.01* |
| 2019 | 0.09 | 3.41 | <0.01* |
| 2020 | 0.08 | 3.31 | <0.01* |
| 2021 | 0.06 | 2.85 | <0.01* |
| 2022 | 0.07 | 2.89 | <0.01* |

**Table S2** LISA of TB notification rate in Hainan Province

| Regions | Local indicators of Spatial association (LISA) | | | | | | | | | |
| --- | --- | --- | --- | --- | --- | --- | --- | --- | --- | --- |
|  | 2013 | 2014 | 2015 | 2016 | 2017 | 2018 | 2019 | 2020 | 2021 | 2022 |
| Baisha | - | - | - | - | - | L-H | H-H | H-H | H-H | H-H |
| Baoting | H-H | - | - | H-H | H-H | H-H | H-H | H-H | H-H | H-H |
| Changjiang | - | - | L-H | L-H | H-H | H-H | - | H-H | - | - |
| Chengmai | - | - | - | - | - | - | - | - | - | - |
| Danzhou | - | - | - | - | - | - | - | - | - | - |
| Dingan | - | - | - | - | - | - | - | - | - | - |
| Dongfang | - | - | - | - | - | H-H | L-H | H-H | L-H | L-H |
| Haikou | - | - | - | - | - | - | - | - | - | - |
| Ledong | - | H-H | H-H | H-H | H-H | H-H | H-H | H-H | H-H | H-H |
| Lingao | - | - | - | - | - | - | - | - | - | - |
| Lingshui | H-H | H-H | - | H-H | - | - | H-H | H-H | H-H | H-H |
| Qionghai | L-H | - | - | - | - | - | - | - | - | - |
| Qiongzhong | H-H | L-H | - | - | - | H-H | H-H | H-H | H-H | H-H |
| Sanya | L-H | L-H | L-H | L-H | L-H | L-H | L-H | L-H | L-H | H-H |
| Tunchang | - | - | - | - | - | - | - | - | - | - |
| Wanning | H-H | - | - | - | - | - | - | - | H-H | - |
| Wenchang | - | - | - | - | - | - | - | - | - | - |
| Wuzhishan | L-H | H-H | L-H | H-H | L-H | H-H | H-H | H-H | H-H | H-H |

Note: No case was recorded in Sansha city. H-H: High-High Cluster, L-H: Low-High Outlier, -: Not significant.

***Population distribution***


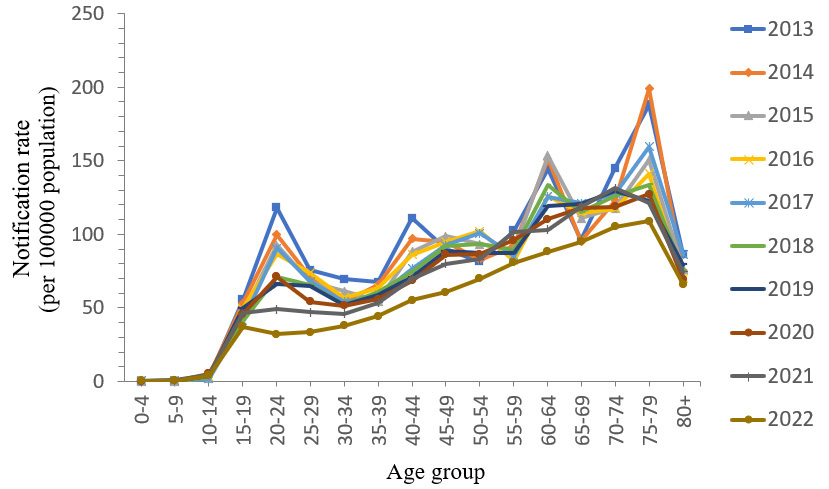


**Figure S4** Changes in the notification rate of TB with age in Hainan Province from 2013 to 2022

**Table S3** TB cases with different population characteristics in Hainan Province from 2013 to 2022

| Characteristics | 2013 | 2014 | 2015 | 2016 | 2017 | 2018 | 2019 | 2020 | 2021 | 2022 | 2013–2022 |
| --- | --- | --- | --- | --- | --- | --- | --- | --- | --- | --- | --- |
| **gender** |  |  |  |  |  |  |  |  |  |  |  |
| male | 5680 | 5280 | 5237 | 5191 | 5073 | 4973 | 4755 | 4664 | 4456 | 3575 | 48,884 |
| female | 1782 | 1693 | 1616 | 1585 | 1599 | 1459 | 1522 | 1440 | 1375 | 1087 | 15,158 |
| **occupation** |  |  |  |  |  |  |  |  |  |  |  |
| farmer | 4820 | 4652 | 4847 | 4507 | 4632 | 4584 | 4300 | 4100 | 3970 | 3010 | 43,422 |
| unemployed | 974 | 797 | 652 | 805 | 603 | 447 | 492 | 583 | 576 | 638 | 6567 |
| retired | 218 | 241 | 186 | 220 | 208 | 213 | 269 | 280 | 324 | 237 | 2396 |
| student | 220 | 179 | 179 | 191 | 175 | 203 | 272 | 285 | 278 | 233 | 2215 |
| worker | 380 | 249 | 189 | 205 | 166 | 178 | 130 | 121 | 112 | 85 | 1815 |
| others | 850 | 855 | 800 | 848 | 888 | 807 | 814 | 735 | 571 | 459 | 7627 |
| **nationality** |  |  |  |  |  |  |  |  |  |  |  |
| Han | 5940 | 5509 | 5316 | 5160 | 4974 | 4661 | 4481 | 4438 | 4271 | 3353 | 48,103 |
| Li | 1467 | 1419 | 1461 | 1532 | 1619 | 1698 | 1733 | 1592 | 1476 | 1236 | 15,233 |
| others | 55 | 45 | 76 | 84 | 79 | 73 | 63 | 74 | 84 | 73 | 706 |

**Migrant features of TB cases**


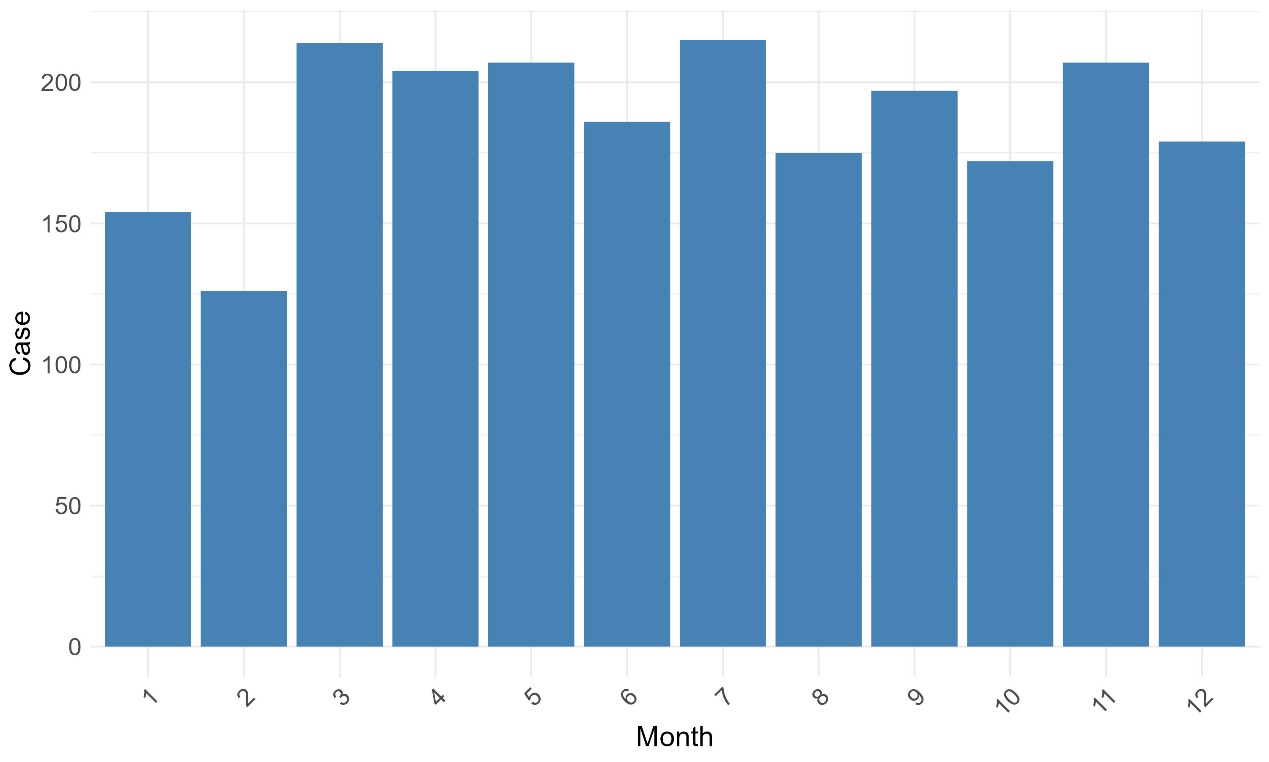


**Figure S5** Time distribution of TB cases from outside Hainan Province


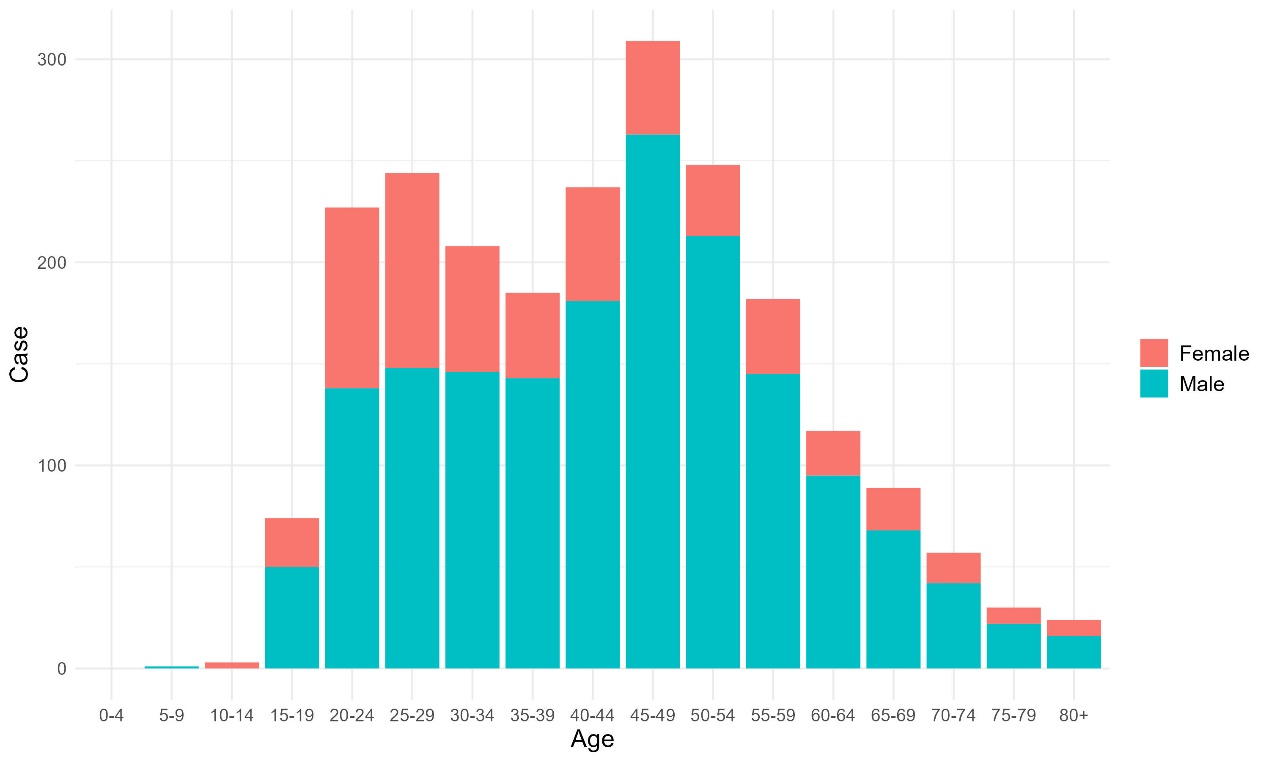


**Figure S6** Age and gender distribution of migrant TB cases outside Hainan Province


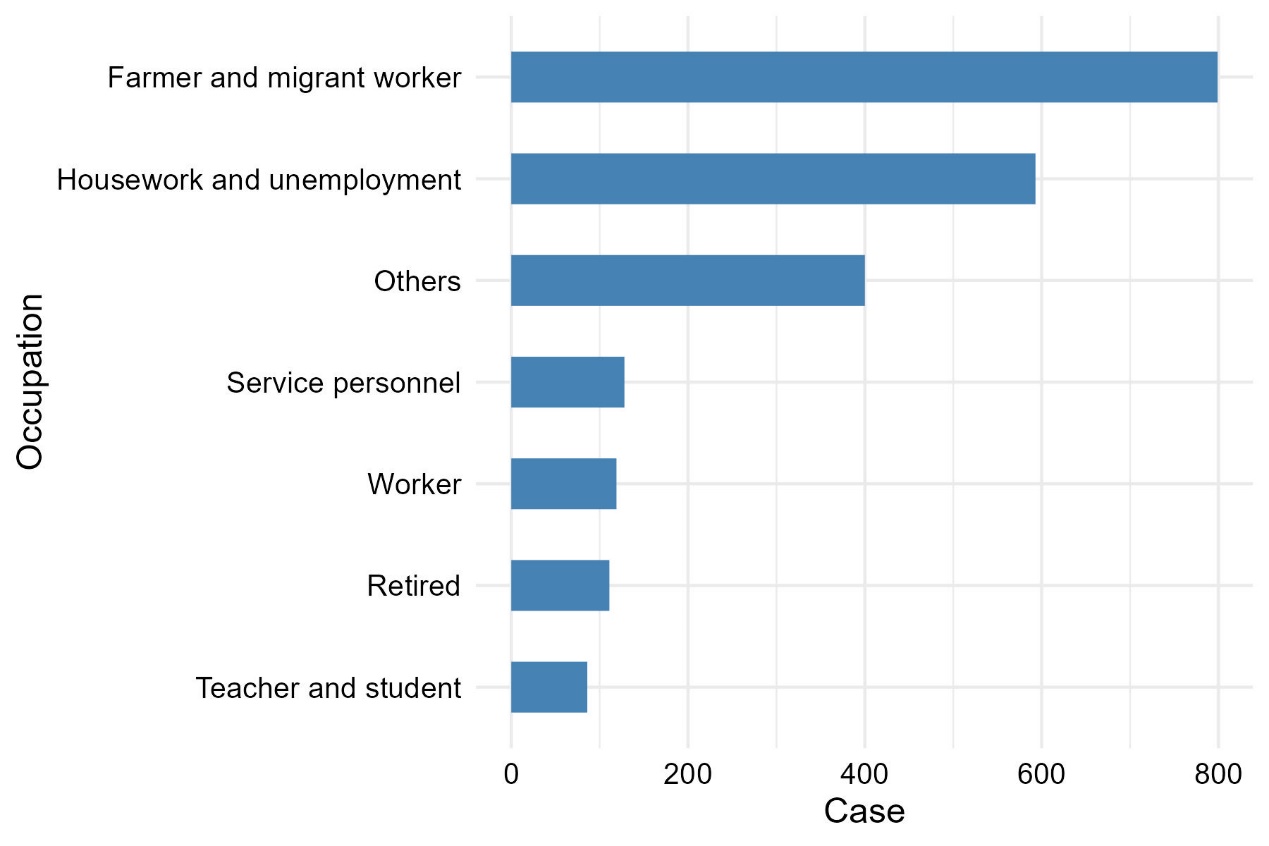


**Figure S7** Occupational distribution of migrant TB cases outside Hainan Province

**Finding patterns of TB cases**


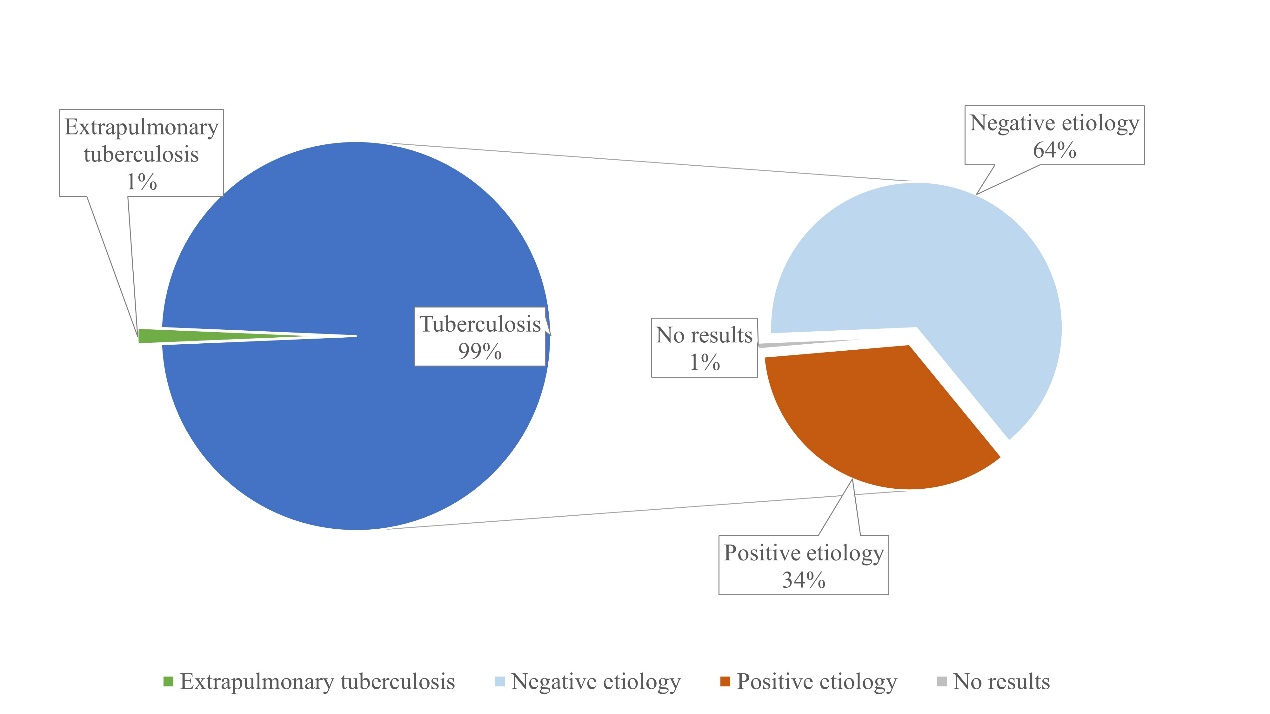


**Figure S8** Notification distribution of TB patients in Hainan Province from 2013 to 2022

**Table S4** Patient delay situation of TB patients in Hainan Province

| Characteristics | n (%) | patient delay days (Q1，Q3) | patient delay ratio (%)  (n) |
| --- | --- | --- | --- |
| **Overall** | 63822 (100) | 30 (10, 61) | 66.78 (42618) |
| **Gender** |  |  |  |
| female | 15,106 (23.67) | 31 (9, 61) | 66.86 (10100) |
| male | 48,716 (76.33) | 30 (10, 61) | 66.75 (32518) |
| **Occupation** |  |  |  |
| famer | 43,274 (67.80) | 31 (11, 61) | 68.69 (29727) |
| worker | 1813 (2.84) | 31 (9, 61) | 68.45 (1241) |
| unemployed | 6542 (10.25) | 30 (6, 62) | 62.24 (4072) |
| student | 2210 (3.46) | 17 (5, 38.75) | 53.85 (1190) |
| retired | 2388 (3.74) | 30 (9, 62) | 66.16 (1580) |
| others | 7595 (11.90) | 30 (7, 61) | 63.30 (4908) |
| **Age** | |  |  |
| ≤ 24 | 7453 (11.68) | 24 (6, 46) | 59.49 (4434) |
| 25-44 | 20,549 (32.20) | 30 (9, 61) | 66.04 (13571) |
| 45-54 | 24,270 (38.03) | 31 (11, 62) | 69.65 (16905) |
| ≥65 | 11,550 (18.10) | 30 (10, 61) | 66.74 (7708) |
| **Nationality** | |  |  |
| Han | 47,931 (75.10) | 29 (8, 55) | 63.69 (30527) |
| Li | 15,189 (23.80) | 32 (15, 65) | 76.23 (11578) |
| others | 702 (1.10) | 33 (14, 73.75) | 73.08 (513) |

**Spatio-temporal analysis of TB**

**Table S5** Influencing factors for TB in Hainan Province from 2013 to 2022

| Regions | Proportion of rural population (%) | | | GDP per capita (10,000 Yuan) | | | Number of medical institutions per 10,000 people | | | Number of health personnel per 10,000 people | | |
| --- | --- | --- | --- | --- | --- | --- | --- | --- | --- | --- | --- | --- |
|  | P_25_ | P_50_ | P_75_ | P_25_ | P_50_ | P_75_ | P_25_ | P_50_ | P_75_ | P_25_ | P_50_ | P_75_ |
| Baisha | 63.32 | 64.09 | 67.84 | 2.39 | 2.82 | 3.39 | 7.95 | 8.04 | 8.12 | 66.21 | 71.23 | 76.50 |
| Baoting | 60.83 | 63.71 | 65.75 | 2.62 | 3.09 | 3.62 | 6.86 | 6.93 | 7.19 | 62.29 | 64.72 | 72.02 |
| Changjiang | 44.52 | 45.27 | 47.69 | 4.23 | 5.14 | 5.39 | 6.27 | 6.66 | 6.97 | 74.54 | 78.79 | 84.00 |
| Chengmai | 40.53 | 44.15 | 51.46 | 5.06 | 5.99 | 6.89 | 7.00 | 7.35 | 7.99 | 57.12 | 61.46 | 74.26 |
| Danzhou | 44.55 | 45.98 | 48.22 | 4.73 | 5.54 | 6.53 | 4.80 | 4.95 | 5.10 | 68.87 | 69.40 | 75.96 |
| Dingan | 53.56 | 55.34 | 58.92 | 2.65 | 3.20 | 3.65 | 7.13 | 7.28 | 7.91 | 56.92 | 61.66 | 67.28 |
| Dongfang | 44.44 | 52.45 | 55.62 | 3.47 | 3.93 | 4.40 | 7.58 | 7.89 | 8.42 | 52.35 | 58.08 | 65.87 |
| Haikou | 18.98 | 21.58 | 22.63 | 5.35 | 6.24 | 6.98 | 3.87 | 4.27 | 4.76 | 123.31 | 130.43 | 134.30 |
| Ledong | 60.71 | 62.78 | 66.76 | 2.28 | 2.60 | 3.17 | 5.51 | 5.66 | 6.93 | 53.52 | 57.25 | 72.87 |
| Lingao | 50.83 | 54.35 | 57.55 | 3.36 | 3.98 | 4.70 | 4.72 | 4.85 | 5.39 | 45.87 | 47.98 | 54.89 |
| Lingshui | 53.10 | 54.84 | 57.38 | 3.71 | 4.68 | 5.41 | 4.72 | 4.80 | 5.37 | 55.80 | 62.95 | 72.61 |
| Qionghai | 48.14 | 50.15 | 53.36 | 4.10 | 4.99 | 5.52 | 5.44 | 5.67 | 5.94 | 69.90 | 73.32 | 104.09 |
| Qiongzhong | 59.38 | 60.97 | 66.41 | 2.27 | 2.71 | 3.24 | 6.51 | 7.27 | 8.11 | 84.78 | 87.97 | 93.02 |
| Sanya | 23.82 | 25.59 | 28.77 | 5.97 | 6.92 | 7.92 | 4.91 | 5.02 | 5.59 | 89.65 | 96.58 | 101.07 |
| Tunchang | 52.71 | 54.46 | 55.87 | 2.27 | 2.77 | 3.40 | 6.23 | 6.61 | 7.33 | 55.71 | 57.11 | 63.11 |
| Wanning | 48.86 | 51.12 | 55.15 | 3.02 | 3.74 | 4.25 | 7.84 | 8.04 | 8.52 | 55.89 | 61.23 | 71.11 |
| Wenchang | 40.81 | 46.75 | 49.35 | 3.25 | 3.88 | 4.63 | 5.27 | 5.48 | 5.73 | 53.26 | 55.54 | 59.55 |
| Wuzhishan | 41.98 | 44.52 | 46.75 | 2.16 | 2.64 | 3.13 | 10.61 | 10.68 | 10.87 | 119.89 | 133.78 | 149.97 |

Note: No case was recorded in Sansha city.

**Table S6** Results from GTWR model analysis

| Variables | Coefficient estimates | | |
| --- | --- | --- | --- |
|  | P_25_ | Median | P_75_ |
| Intercept | 45.27 | 113.57 | 226.57 |
| Proportion of rural population (%) | -0.94 | -0.13 | 0.45 |
| GDP per capita (Yuan) | -1.70×10^-3^ | -6.52×10^-4^ | -2.84×10^-5^ |
| Number of medical institutions per 10,000 population | -7.20 | 0.40 | 4.76 |
| Number of health personnel per 10,000 population | -0.56 | -0.14 | 0.28 |
| **Model diagnostic information** |  |  |  |
| R^2^ | 0.86 |  |  |
| Adjusted R^2^ | 0.85 |  |  |
| AICc | 1514.32 |  |  |
| RMSE | 11.46 |  |  |

**TB Elimination in Hainan Province**

**
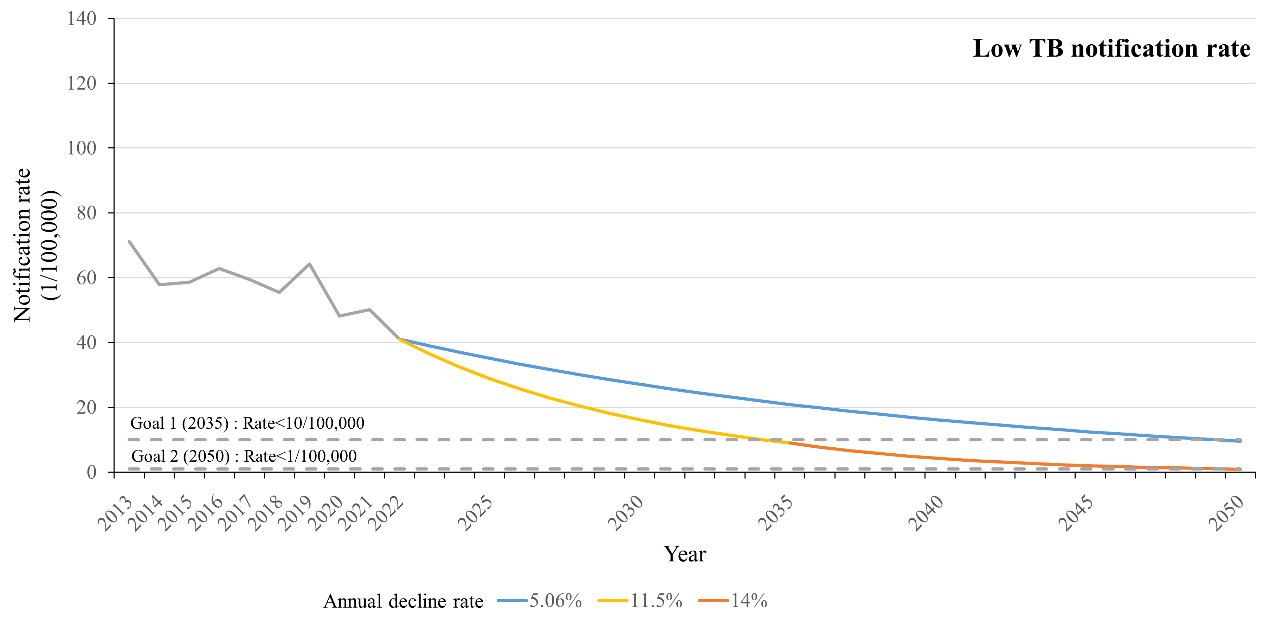
**

**Figure S9** TB notification rate in regions with low TB notification rate last decade and future prediction (***Note:*** Blue was the average annual decline rate in last decade, yellow and orange were the estimated annual decline rates to meet the TB elimination goal. The estimated annual decline rate before 2035: 11.5%; The estimated annual decline rate before 2050: 14%)

**
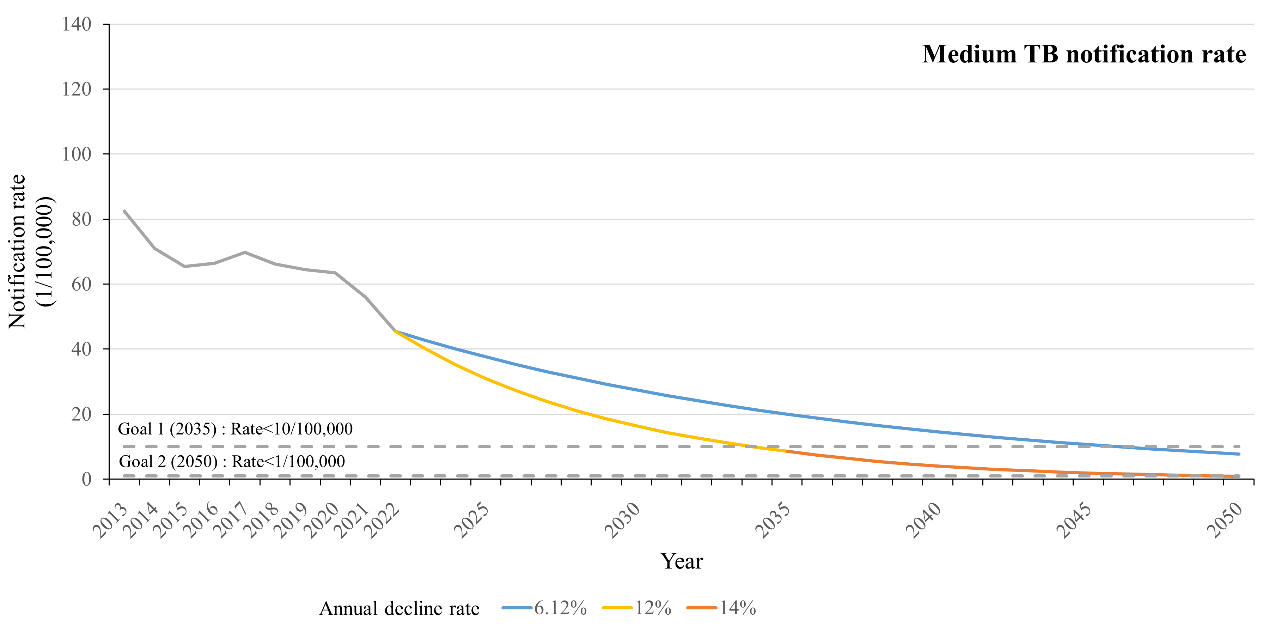
**

**Figure S10** TB notification rate in regions with medium TB notification rate last decade and future prediction (***Note:*** Blue was the average annual decline rate in last decade, yellow and orange were the estimated annual decline rates to meet the TB elimination goal. The estimated annual decline rate before 2035: 12%; The estimated annual decline rate before 2050: 14%)

**
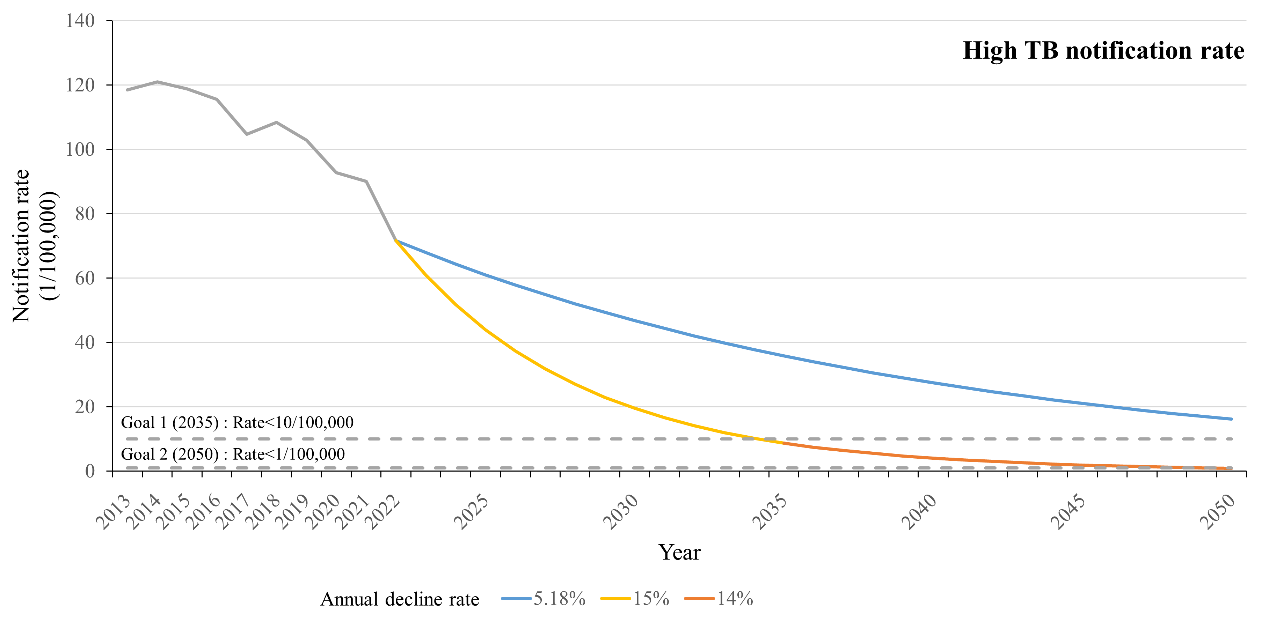
**

**Figure S11** TB notification rate in regions with high TB notification rate last decade and future prediction (***Note:*** Blue was the average annual decline rate in last decade, yellow and orange were the estimated annual decline rates to meet the TB elimination goal. The estimated annual decline rate before 2035: 15%; The estimated annual decline rate before 2050: 14%)

**Table S7** Main risk factors influencing the prevalence of TB in different cities and counties

| Counties with higher notification rate | Main influencing factors |
| --- | --- |
| Lingshui | B, C |
| Ledong | B, C, D |
| Dongfang | B, C, D |
| Baoting | B, C |
| Qiongzhong | A, C |
| Wanning | A, C, D |
| Wuzhisha | B, C |

Note: A: Proportion of rural population; B: GDP per capita; C: Number of medical institutions per 10,000 population; D: Number of health personnel per 10,000 population.
